# A subset of broadly responsive Type III taste cells contribute to the detection of bitter, sweet and umami stimuli

**DOI:** 10.1371/journal.pgen.1008925

**Published:** 2020-08-13

**Authors:** Debarghya Dutta Banik, Eric D. Benfey, Laura E. Martin, Kristen E. Kay, Gregory C. Loney, Amy R. Nelson, Zachary C. Ahart, Barrett T. Kemp, Bailey R. Kemp, Ann-Marie Torregrossa, Kathryn F. Medler

**Affiliations:** 1 Department of Biological Sciences, University at Buffalo, Buffalo, New York, United States of America; 2 Department of Psychology, University at Buffalo, Buffalo, New York, United States of America; 3 Center for Ingestive Behavior Research, University at Buffalo, Buffalo, New York, United States of America; University of Colorado School of Medicine, UNITED STATES

## Abstract

Taste receptor cells use multiple signaling pathways to detect chemicals in potential food items. These cells are functionally grouped into different types: Type I cells act as support cells and have glial-like properties; Type II cells detect bitter, sweet, and umami taste stimuli; and Type III cells detect sour and salty stimuli. We have identified a new population of taste cells that are broadly tuned to multiple taste stimuli including bitter, sweet, sour, and umami. The goal of this study was to characterize these broadly responsive (BR) taste cells. We used an IP_3_R3-KO mouse (does not release calcium (Ca^2+^) from internal stores in Type II cells when stimulated with bitter, sweet, or umami stimuli) to characterize the BR cells without any potentially confounding input from Type II cells. Using live cell Ca^2+^ imaging in isolated taste cells from the IP_3_R3-KO mouse, we found that BR cells are a subset of Type III cells that respond to sour stimuli but also use a PLCβ signaling pathway to respond to bitter, sweet, and umami stimuli. Unlike Type II cells, individual BR cells are broadly tuned and respond to multiple stimuli across different taste modalities. Live cell imaging in a PLCβ3-KO mouse confirmed that BR cells use this signaling pathway to respond to bitter, sweet, and umami stimuli. Short term behavioral assays revealed that BR cells make significant contributions to taste driven behaviors and found that loss of either PLCβ3 in BR cells or IP_3_R3 in Type II cells caused similar behavioral deficits to bitter, sweet, and umami stimuli. Analysis of c-Fos activity in the nucleus of the solitary tract (NTS) also demonstrated that functional Type II and BR cells are required for normal stimulus induced expression.

## Author summary

We use our taste system to decide if we are going to consume or reject a potential food item. This is critical for survival, as we need energy to live but also need to avoid potentially toxic compounds. Therefore, it is important to understand how the taste cells in our mouth detect the chemicals in food and send a message to our brain. Signals from the taste cells form a code that conveys information about the nature of the potential food item to the brain. How this taste coding works is not well understood. Currently, it is thought that taste cells are primarily selective for each taste stimuli and only detect either bitter, sweet, sour, salt, or umami (amino acids) compounds. Our study describes a new population of taste cells that can detect multiple types of stimuli, including chemicals from different taste qualities. Thus, taste cells can be either selective or generally responsive to stimuli which is similar to the cells in the brain that process taste information. The presence of these broadly responsive taste cells provides new insight into how taste information is sent to the brain for processing.

## Introduction

**Fig 1:**
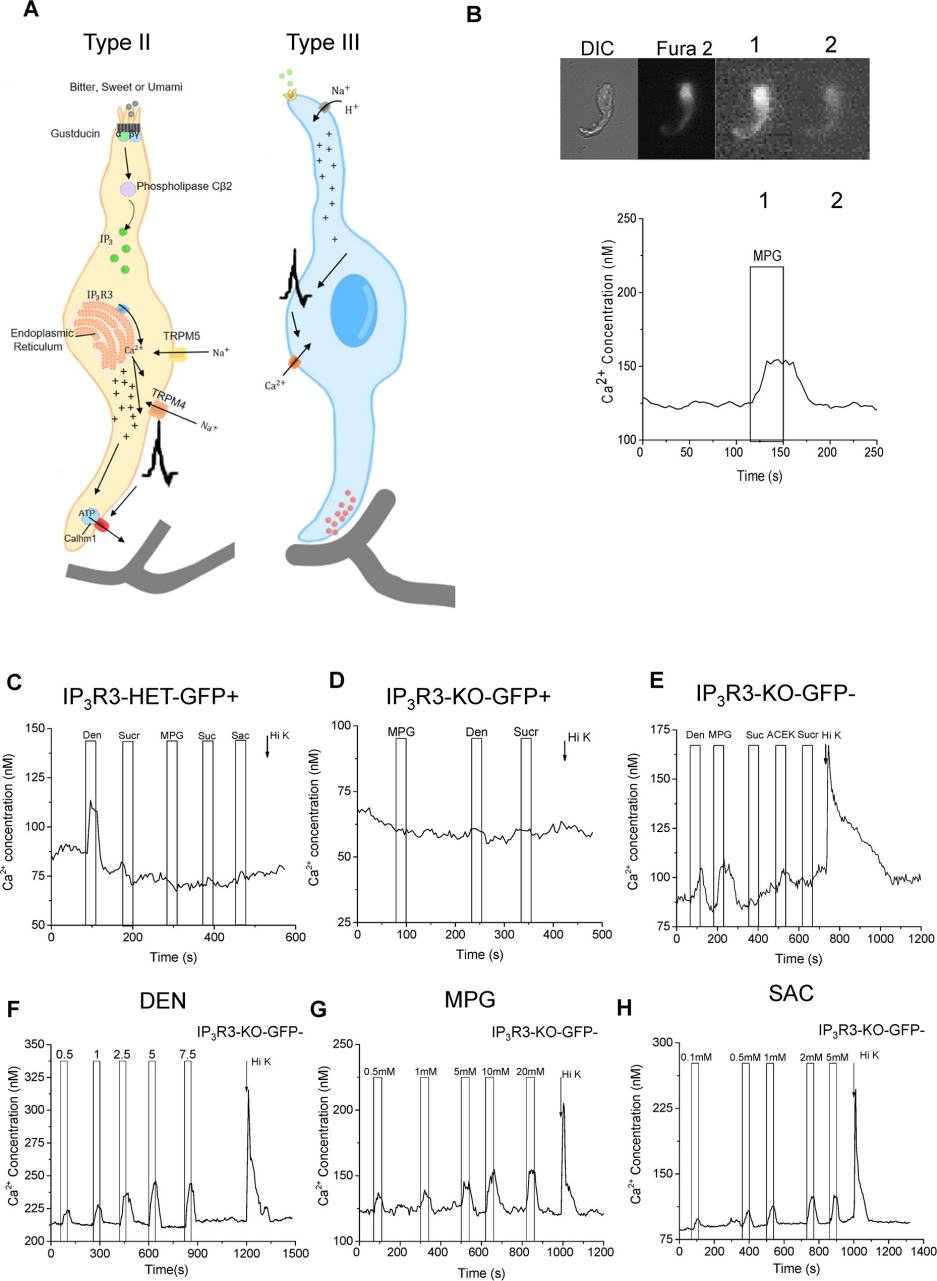
Current taste cell models and experimental setup. A) Current models of the signal transduction pathways in Type II taste cells (left) that respond to bitter, sweet, and umami stimuli as well as Type III taste cells (right) that respond to sour and salty stimuli. B) Example of an isolated taste cell that was stimulated with MPG (10mM). The DIC image is shown first, followed by an image of Fura 2-AM loading. The next two images show the ratio (340/380) at different time points as the cell is stimulated and then recovers to baseline. The corresponding calcium (Ca^2+^) changes are shown in the imaging trace. C) The IP_3_R3-HET-GFP+ cell responded to denatonium, but did not respond to any of the other stimuli applied. D) The IP_3_R3-KO-GFP+ cell did not respond to any stimulus applied. E) The IP_3_R3-KO-GFP- cell responded to denatonium, MPG and 50mM KCl (denoted with arrow, Hi K), identifying it as a BR cell. Concentration gradients for (F) bitter (denatonium, DEN), (G) umami (MPG) and (H) sweet (saccharin, SAC) in the IP_3_R3-KO mouse line. BR cells respond to a wide range of stimulus concentrations.

Chemicals in the oral cavity are detected by taste receptor cells which are grouped together in taste buds found in epithelial specializations called papillae. The current view of taste transduction is that taste buds are comprised of Type I, Type II, and Type III taste receptor cells along with basal cells that are precursors for the differentiated cells [1]. Type I cells are thought to primarily function as support cells and share some characteristics of glia cells [2], while Type II cells detect bitter, sweet, or umami stimuli through the activation of specific GPCRs for each of these taste qualities. Transduction of these taste qualities in Type II cells is due to a single signaling pathway that is comprised of phospholipase Cβ2 (PLCβ2) which activates the inositol 1,4,5-trisphosphate receptor type 3 (IP_3_R3) on the endoplasmic reticulum to cause calcium (Ca^2+^) release [3–5]. This Ca^2+^ release activates the transient receptor potential cation channel subfamily M members 4 and 5 (TRPM4 and TRPM5) which depolarize the cell sufficiently to activate the release of ATP through the calcium homeostasis modulator 1 (CALHM1) channel [4, 6–10]. The expression of PLCβ2, IP_3_R3, and TRPM5 is restricted to Type II taste cells and these cells lack both voltage-gated Ca^2+^ channels (VGCCs) and conventional chemical synapses (model shown in Fig 1A)[11–15]. Type III cells detect sour and salt stimuli through ionotropic receptors that depolarize the cell to activate VGCCs and cause vesicular neurotransmitter release (see Fig 1A) [16–22]. It is currently thought that Type III cells do not respond to bitter, sweet, or umami stimuli.

We previously reported that some taste cells generate Ca^2+^ signals to both denatonium, a bitter stimulus and cell depolarization with 50mM KCl [23]. Since cells expressing VGCCs respond to cell depolarization with a Ca^2+^ signal, these data identified a potentially unique taste cell population that expresses VGCCs but also responds to denatonium [23]. To date, these cells have not been characterized. We hypothesized that there is a subset of Type III taste cells that are responsive to denatonium and are potentially sensitive to multiple bitter, sweet, and umami stimuli. To address this question, we used live cell imaging in an IP_3_R3-KO mouse line to evaluate these cells without Ca^2+^ responses from Type II cells. We identified a subset of Type III cells that respond to bitter, sweet, and/or umami stimuli and determined that these broadly-responsive (BR) taste cells use a PLCβ3 signaling pathway. The loss of PLCβ3 in the BR cells caused significant deficits in subsequent taste perception, supporting the conclusion that these BR cells are important for taste.

## Results

### Bitter, sweet and umami taste evoked responses are still present in IP_3_R3-KO mice

To assess the characteristics of the BR cells, we wished to avoid any potential signaling input from the Type II cells that respond to bitter, sweet, and umami stimuli. IP_3_R3 is part of the canonical signaling pathway in Type II cells that are sensitive to these stimuli [3, 11, 15, 24]. Therefore, we used a transgenic mouse in which GFP replaces the coding region of IP_3_R3 to evaluate the taste evoked signaling in taste receptor cells that lack the ability to release Ca^2+^ via IP_3_R3 [25]. In these mice, GFP labels the cells that should express IP_3_R3 but no longer do so. Initial immunohistochemical analyses found that taste cells from the CV papillae of wild type mice were successfully labeled with anti-IP_3_R3 antibody (n = 3; S1A Fig), while their KO littermates lacked IP_3_R3 labeling but instead had GFP expression (n = 6; S1B Fig). Furthermore, anti-IP_3_R3 labels the GFP expressing cells in IP_3_R3-heterzygous mice (n = 4; S1C Fig); confirming that GFP expression identifies the taste cells that normally express IP_3_R3. To further characterize this mouse, we evaluated the expression of other proteins that are part of this established signaling pathway in Type II cells. The GFP expression in the KO mice co-localized with both PLCβ2 (n = 4; S1D Fig) and gustducin (n = 3; S1E Fig) labeling. As expected, gustducin expression was restricted to the IP_3_R3-GFP-KO cells but not all GFP+ cells had gustducin. There was, however, complete overlap between anti-PLCβ2 labeling and GFP. Co-localization analyses are reported in S1 Fig. Thus, the other components of the signaling pathway normally expressed in Type II cells are intact but these cells no longer have the capacity to release Ca^2+^ in response to bitter, sweet, or umami stimuli.

We also functionally characterized the ability of the taste cells in this mouse to respond to taste stimuli. Measurements were made in isolated individual taste cells to ensure that the measured taste-evoked Ca^2+^ responses were due solely to the activity of the individual taste cells and not cell-to-cell communication (example of a response in an isolated cell in Fig 1B). To functionally characterize the IP_3_R3-GFP-KO mouse, and to confirm that GFP is expressed in Type II cells, we measured the taste-evoked Ca^2+^ responses in GFP+ taste cells from the IP_3_R3-het mouse which express GFP but also have a functional copy of the IP_3_R3 gene. These cells responded to taste stimuli but did not generate a Ca^2+^ signal to 50mM KCl, indicating these cells do not express VGCCs (Fig 1C) and are likely Type II cells. Alternatively, GFP+ cells from the IP_3_R3-KO mouse (lack a functional IP_3_R3 gene) did not produce Ca^2+^ signals when stimulated (Fig 1D). When we targeted the GFP- cells in the IP_3_R3-KO mouse, we identified taste cells that responded to both taste stimuli and 50mM KCl with a Ca^2+^ signal (Fig 1E). These data suggest that these BR taste cells do not require input from Type II cells to respond to bitter, sweet, or umami stimuli.

We characterized BR cells by applying taste stimuli to isolated GFP- taste cells from IP_3_R3-KO mice. Since our goal was to identify all (or most) responsive taste cells, we used the stimulus concentration that generated a maximal Ca^2+^ signal. For all experiments, taste stimuli were applied for 40s (application shown with bar in graphs) and 50mM KCl was applied for 10s (application shown with arrow in graphs). These concentrations were based on our previous control experiments to identify the lowest stimulus concentration that generated the maximal Ca^2+^ signal (examples shown in Fig 1F, 1G, and 1H).

**Fig 2:**
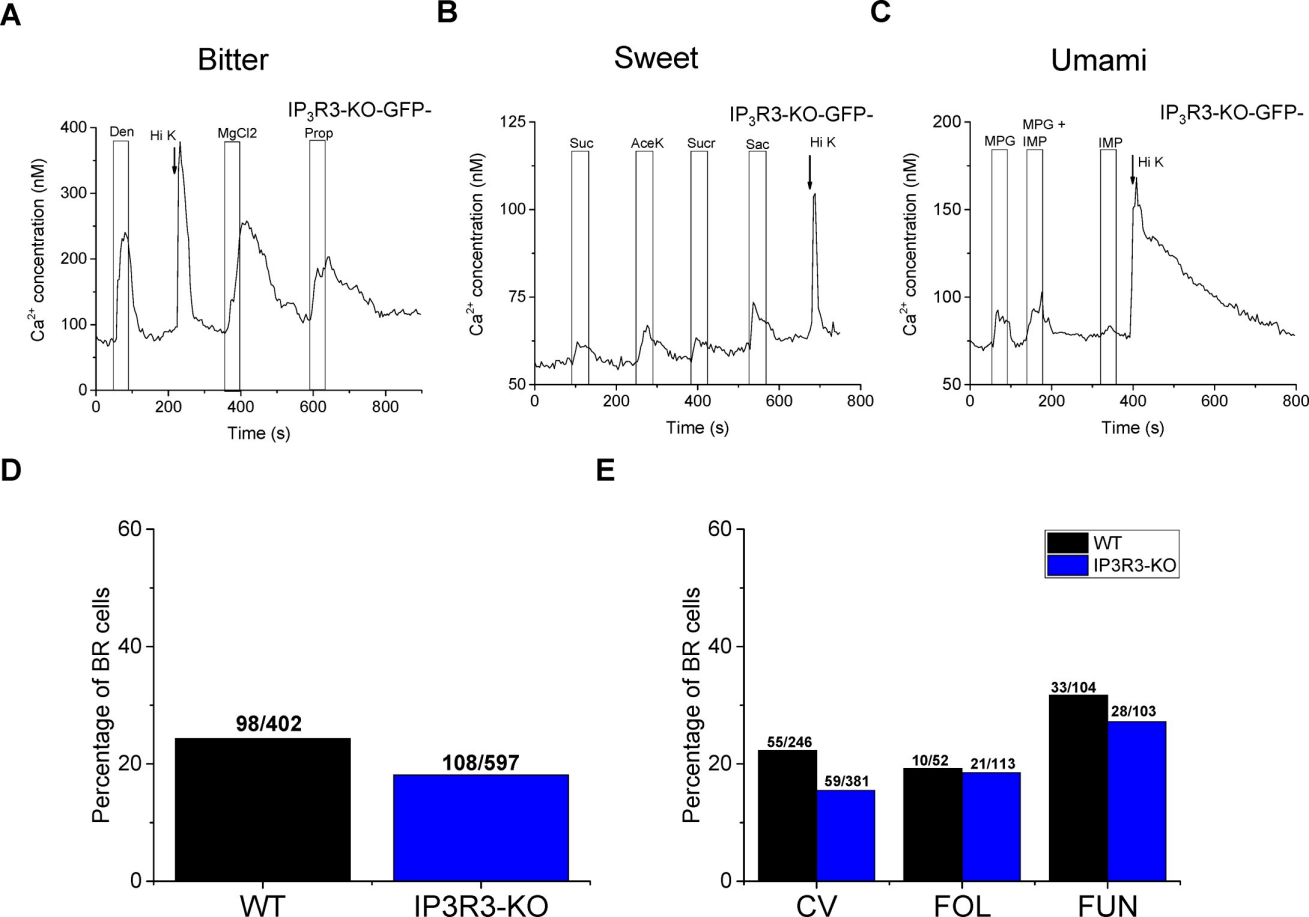
BR taste cells respond to multiple taste stimuli independently of the IP_3_R3 protein expressed in Type II cells. Representative traces of BR taste cells that were stimulated with multiple bitter (A), sweet (B, 20mM Sucralose, Sucr; 50mM Sucrose, Suc), or umami (C) stimuli as well as 50mM KCl (arrow, Hi K). D) Chi square analysis with Yate’s correction for continuity was used to compare the response rate or frequency of evoked Ca^2+^ responses to different taste stimuli between wild type (WT) and IP_3_R3-KO (KO) mice (p = 0.062). E) Chi square analysis with Yate’s correction for continuity was used to compare the response rate or frequency of evoked Ca^2+^ responses by BR cells from wild type (WT) and IP_3_R3-KO (KO) mice for circumvallate (CV, p = 0.09), foliate (Fol, p = 0.89) and fungiform (Fun, p = 0.7) taste cells. Number of responses per number of cells tested are presented in the graphs.

Type III cells are the only known population of taste cells that express VGCCs [26] and are functionally identified in live cell imaging by the ability to respond to cell depolarization with a Ca^2+^ influx through the opening of VGCCs [15, 18, 26–29]. Therefore, if a taste cell responded to 50mM KCl with a Ca^2+^ signal, we concluded that it expresses VGCCs and is a Type III cell. We functionally identified a taste cell as a Type II cell if it responded to a taste stimulus (bitter, sweet, or umami) but did not respond to 50mM KCl. Taste cells were identified as BR cells if they responded to both a taste stimulus and 50mM KCl. We applied multiple bitter, sweet, and umami stimuli + 50mM KCl to isolated taste receptor cells from IP_3_R3-KO mice (Fig 2A, 2B and 2C). We found that BR cells often responded to multiple stimuli, including multiple types of stimuli for each taste quality. To evaluate if the loss of IP_3_R3 affected this population of BR cells, we compared the percentage of BR cells between the WT (n = 105 mice tested) and IP_3_R3-KO mice (n = 115 mice tested). Because taste-evoked response sizes are variable, we focused our analysis on the response rate or frequency of cell responses. A response was recorded if the isolated taste cell had a stable baseline and application of the taste stimulus caused an increase in fluorescence that was at least 2 standard deviations above the baseline fluorescence level. We found no significant differences in the response rate of taste cells that were BR in the IP_3_R3-KO mice compared to WT (Fig 2D). Thus, these data demonstrate that the BR cell responses do not rely on Type II cell responses.

There are three papillae types that house taste buds in different areas of the tongue: circumvallate (CV), foliate (Fol), and fungiform (Fun). We measured the response rate of BR cells for the different papillae types in the WT and IP_3_R3-KO mice and found no significant differences (Fig 2E). Thus, BR cells are present in multiple taste papillae and the loss of IP_3_R3 in Type II cells does not affect the percentage of taste cells that are BR.

### A specific subset of Type III cells is broadly responsive (BR)

**Fig 3:**
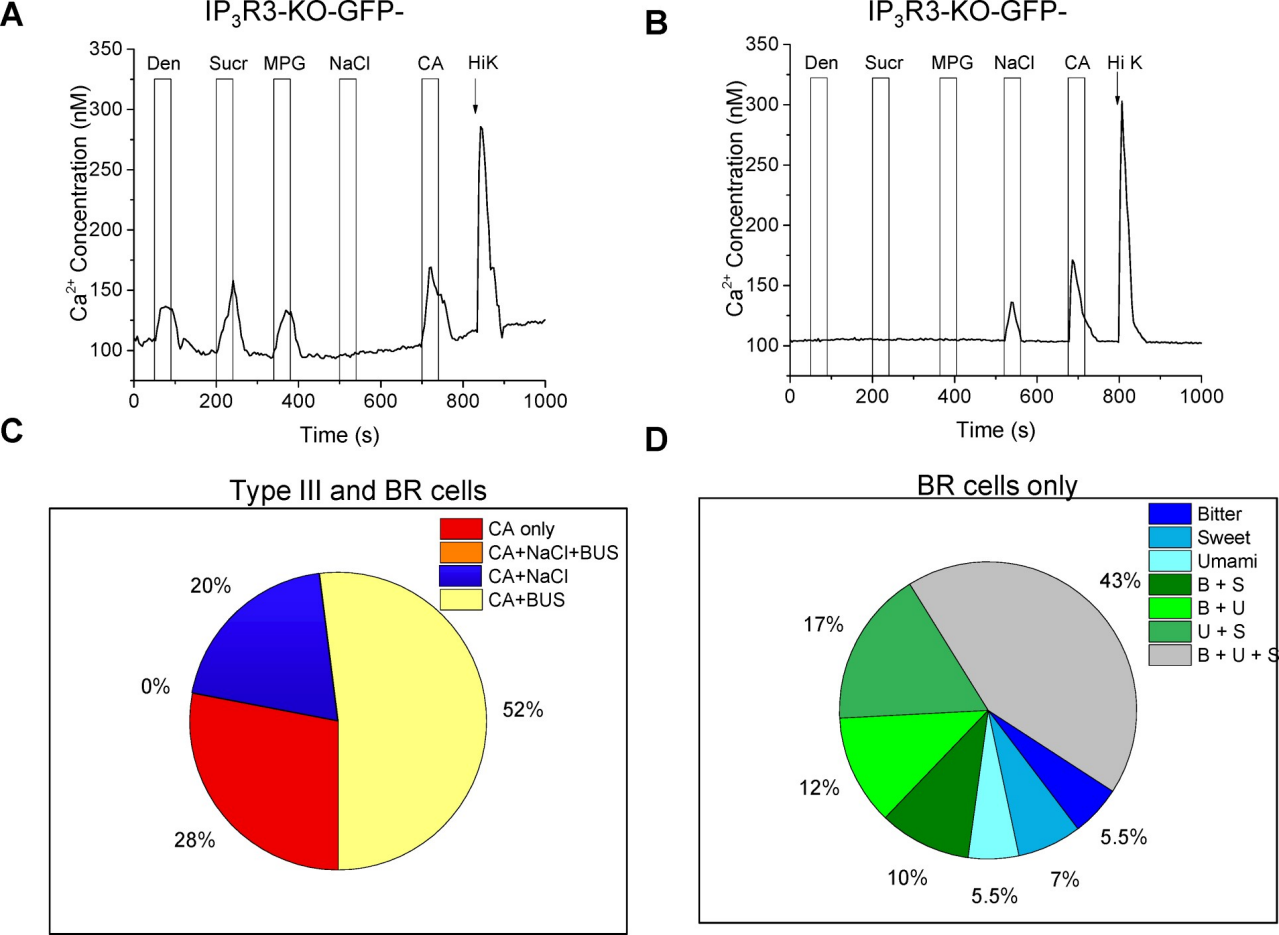
Some Type III cells respond to bitter, sweet and umami stimuli. A) Representative trace of taste cells from IP_3_R3-KO mice that responded to bitter (5mM denatonium, Den), sweet (20mM sucralose, Sucr), umami (10mM monopotassium glutamate, MPG), 50mM citric acid (CA), and 50mM KCl (Hi K). B) Representative trace of a separate subset of Type III cells that responded to 250mM NaCl, 50mM CA and 50mM KCl but were not sensitive to the bitter, sweet, umami stimuli tested. C) Summary of taste cells from IP_3_R3-KO mice that responded to 50mM KCl and CA with Ca^2+^ signals (n = 65), 28% only responded to CA (n = 18), 20% responded to CA and NaCl (n = 13), and 52% responded to CA and bitter, sweet, and/or umami stimuli (B,U,S, n = 34) while no cells responded to all taste stimuli. D) A summary of the response profiles for a larger group of BR Type III cells (n = 127) that either responded to bitter (n = 7), sweet (n = 9), umami (n = 7), bitter + sweet (n = 13), bitter + umami (n = 15), sweet + umami (n = 21), or bitter, sweet + umami (n = 55).

The current understanding is that all Type III cells are sour sensitive and a subset of these sour-sensitive Type III cells are also salt sensitive [16, 18, 29]. To confirm that the BR taste cells are Type III cells, we included sour (citric acid, CA) and salt (NaCl) stimuli in some experiments (Fig 3). In these experiments, we used a representative stimulus for bitter (5mM Den), sweet (20mM sucralose, Sucr), and umami (10mM MPG). All of the taste cells (from IP_3_R3-KO mice) that responded to 50mM KCl responded to citric acid (CA) with a Ca^2+^ signal while some of these cells also responded to bitter (B), sweet (S), and/or umami (U) stimuli with a Ca^2+^ signal (Fig 3A, 3C and 3D). A representative imaging trace is shown in Fig 3A, with the summary of the Type III responses shown in Fig 3C. We did not detect any Type III cells that responded to all 5 taste qualities (Fig 3B and 3C, orange region). However, in addition to the Type III cells that responded to either sour (CA, red region) or sour and salty (CA + NaCl, blue region) there was another subset of Type III cells (52%, yellow region) that responded to sour and at least one bitter, sweet, and/or umami (B, U, S) stimulus. To better understand these BR cells, we increased our sample size and measured the distribution of the taste responses to bitter, sweet, and umami stimuli within these cells (Fig 3D). Unlike the Type II cells, few BR cells responded to a single taste quality (bitter, sweet, and umami were 5.5, 7 and 5.5% respectively, blue regions) but were instead most likely to respond to two or three taste qualities in addition to responding to 50 mM KCl. 39% of the cells responded to two stimuli (green regions) while 43% responded to all three (gray region).

Control experiments using the GAD67-GFP mice as a cellular marker to identify a subset of Type III taste cells [30–32] found that both GAD67-GFP positive and negative taste cells responded to taste stimuli + 50mM KCl with Ca^2+^ signals (S2A and S2B Fig). Approximately 69% (n = 98/143 cells tested) of the GAD67-GFP positive cells responded to a bitter, sweet, or umami stimulus as well as 50mM KCl. BR cells were also identified in C57BL/6 mice (S2C Fig) which agrees with a separate study that reported some Type III cells from C57BL/6 mice responded to bitter stimuli [18]. Together these data suggest that these BR Type III taste cells are present in taste cells in multiple mouse lines.

### BR cells use a PLCβ signaling pathway to respond to bitter, sweet, and/or umami stimuli

**Fig 4:**
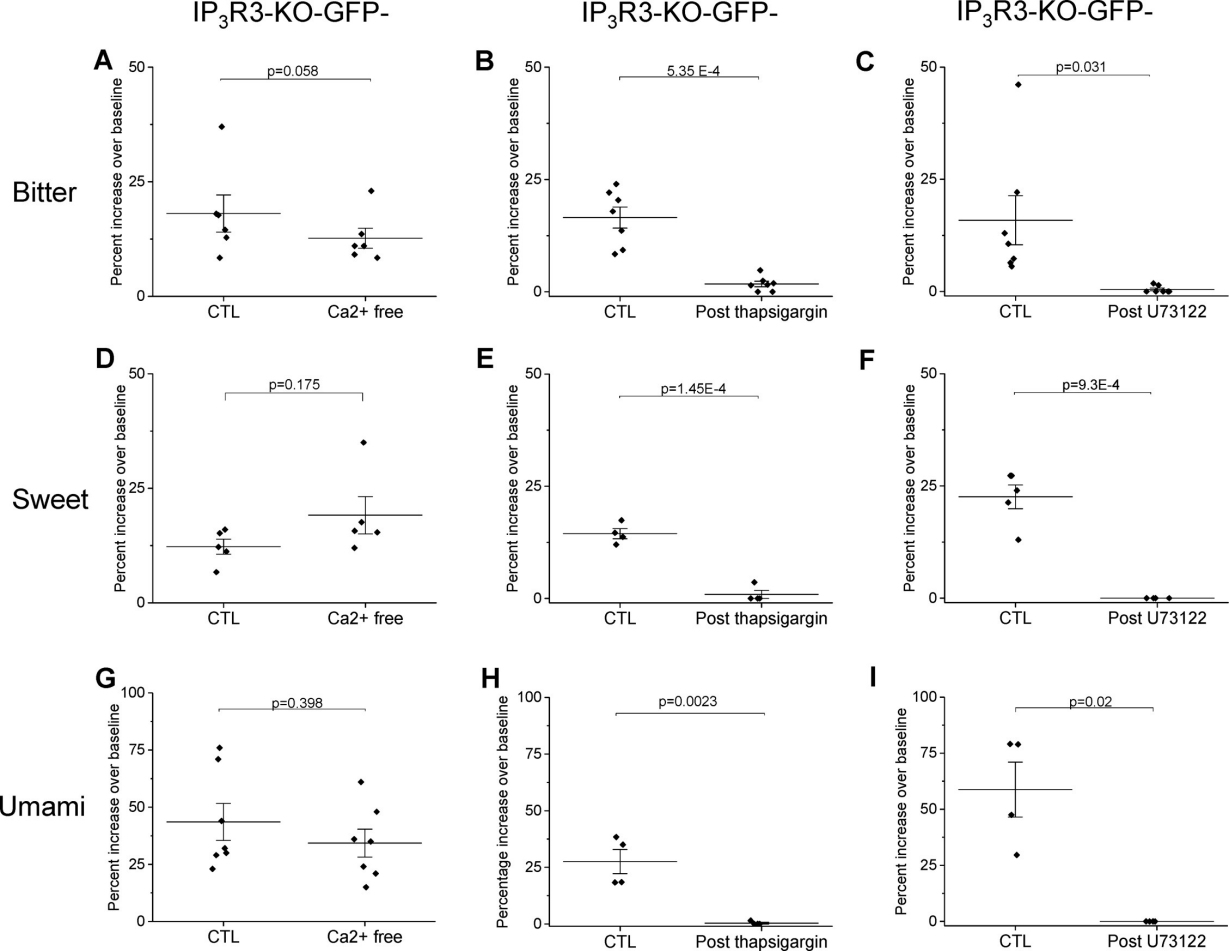
Taste-evoked Ca^2+^ release in BR cells from IP_3_R3-KO mice is dependent upon PLC activity and Ca^2+^ release from internal stores. A) Bitter-evoked taste responses (5mM denatonium, n = 6) persist in the absence of extracellular calcium (Ca^2+^-free) and are abolished by the SERCA pump inhibitor thapsigargin (B, n = 7) as well as the PLC blocker U73122 (C, n = 7). Similar results were obtained for sweet stimuli (20mM sucralose, n = 6) in Ca^2+^-free (D), thapsigargin (E, n = 4) and U73122 (F, n = 6) as well as for umami stimuli (10mM MPG) in Ca^2+^-free (G, n = 7), thapsigargin (H, n = 4) and U73122 (I, n = 4). Representative data for each experiment are shown in S3 Fig. Comparisons of the response amplitudes for each taste stimulus found no significant differences in the control responses between experiments (One way ANOVA, Den, p = 0.933; Suc, p = 0.623; MPG, p = 0.134).

To identify the type of signaling pathway that generates the taste responses in the BR cells, we analyzed the amplitudes of the responses to bitter (5mM Den), sweet (20mM sucralose), and umami (10mM MPG) stimuli in the IP_3_R3-KO mouse. We found that the size of the responses to these stimuli did not significantly change in Ca^2+^-free Tyrode’s, indicating these signals depend on Ca^2+^ release from internal stores (Fig 4A, 4D and 4G). We confirmed these results by applying thapsigargin to disrupt the internal Ca^2+^ stores which abolished the taste evoked Ca^2+^ signals (Fig 4B, 4E and 4H). These responses were also eliminated when the general PLC blocker U73122 was applied (Fig 4C, 4F and 4I), indicating that these taste evoked Ca^2+^ responses in the BR are due to the activation of a PLC signaling pathway. Representative traces for each experimental condition are shown in S3 Fig.

### PLCβ3 expression in peripheral taste cells

To identify other PLCβ isoforms expressed in taste receptor cells, we analyzed our previously published RNAseq data that was collected from a pool of isolated taste cells from 5 mice for each biological repeat [33]. We found that PLCβ2 and PLCβ3 were expressed at comparable levels and were the predominant PLCβ isoforms present in taste cells. Since PLCβ2 is only expressed in Type II cells, we investigated the possibility that PLCβ3 is expressed in peripheral taste cells. Immunohistochemical experiments in the IP_3_R3-KO mouse revealed strong PLCβ3 labeling that was separate from the Type II cells (identified with GFP expression, S4A Fig). These data were confirmed by examining anti-PLCβ3 labeling in the TRPM5-GFP mouse as well as co-labeling with PLCβ2 in C57BL/6 mice (additional Type II cell markers, S4B and S4C Fig). Co-labeling with the Type I taste marker NTPDase2 found that PLCβ3 is not expressed in Type I cells (S4D Fig). We next determined if PLCβ3 is expressed in Type III cells using the GAD67-GFP mouse as well as SNAP-25, a synaptic protein expressed in Type III taste cells [15, 32, 34]. Immunohistochemical analysis found some co-expression of PLCβ3 with GAD67-GFP expression (S4E Fig) as well as some overlap with the SNAP25 expression in C57BL/6 mice (S4F Fig) supporting the conclusion that PLCβ3 is expressed in some Type III cells. Colocalization analyses are shown in S4G Fig.

We also used qPCR to measure the relative amounts of mRNA for PLCβ3 and PLCβ2 in the different taste papillae (S4H and S4I Fig) and found that both PLCβ2 and PLCβ3 are expressed in CV, Fol, and Fun papillae. Therefore, both PLCβ2 and PLCβ3 are expressed in peripheral taste cells and PLCβ3 is expressed in a subset of Type III cells but not either Type I or Type II taste cells.

### PLCβ3 contributes to taste evoked signaling in a subset of Type III cells

**Fig 5:**
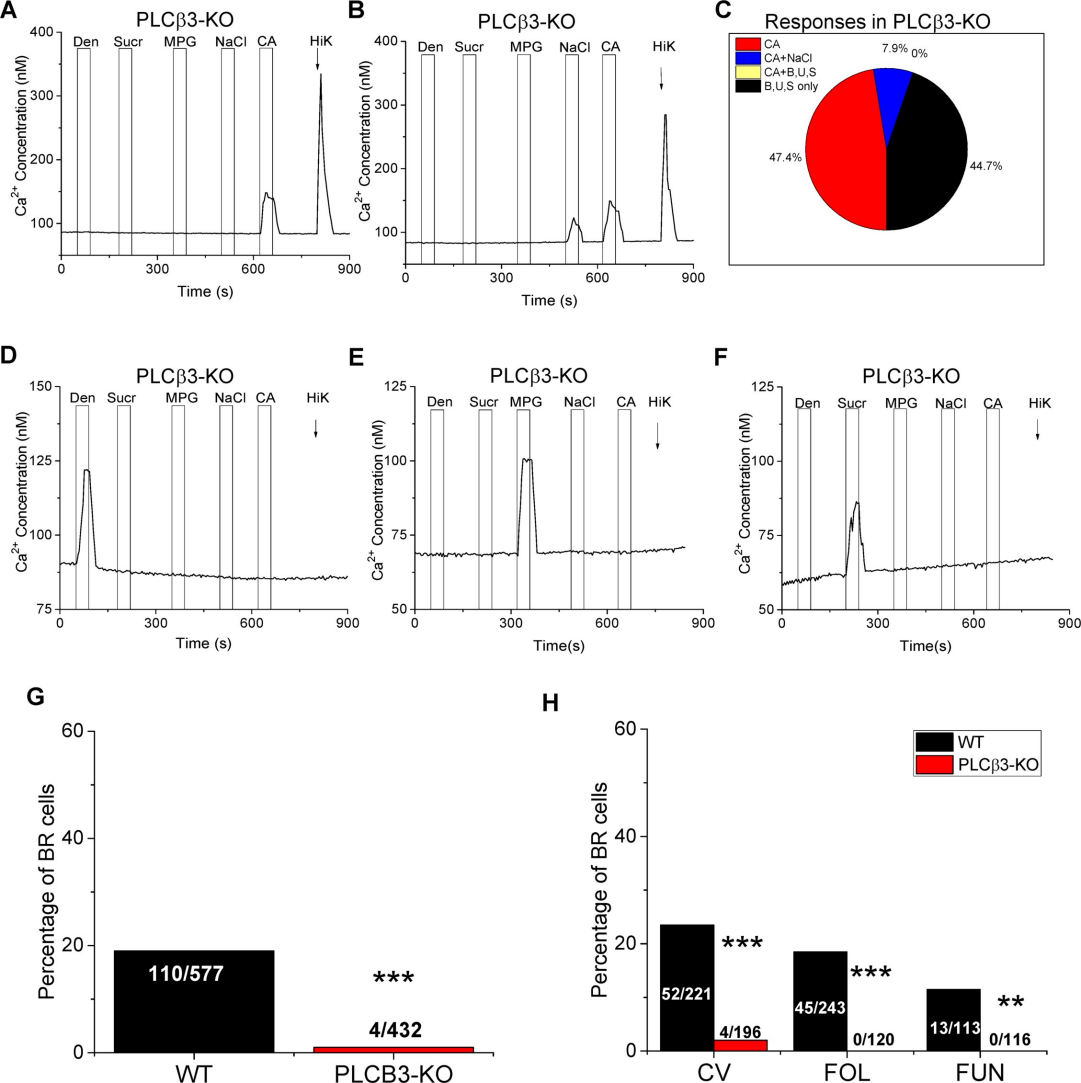
PLCβ3 is required for the detection of bitter, sweet and umami stimuli in BR cells. A) A representative trace from PLCβ3-KO taste cells that responded to 50mM citric acid (CA) and KCl (50mM, Hi K) but did not respond to bitter (5mM denatonium, Den), sweet (20mM sucralose, Sucr), or umami (10mM MPG) stimuli. B) A representative trace from a separate subset of cells that responded to 250mM NaCl, CA, and KCl but were not sensitive to the bitter, sweet, or umami stimuli tested. C) Summary of responsive taste cells from PLCβ3-KO mice (n = 38): CA (n = 18); CA +NaCl (n = 3); CA and bitter, sweet, and/or umami stimuli (B, U, S) (n = 0); and B, U, or S only (n = 17). Representative taste-evoked responses for bitter (D), sweet (E), and umami (F) in the PLCβ3-KO mice. None of these cells responded to CA or KCl. G) Chi square analysis with Yate’s correction for continuity was used to compare the response rate or frequency of BR cells in wild type (n = 53) and PLCβ3-KO (n = 68) mice (***, p<0.001) for a larger number of cells. H) The percentage of BR taste cells from the CV, Fol, and Fun papillae of the PLCβ3-KO mice and WT mice were compared (***, p<0.001 for CV and Fol; **, p = 0.011 for Fun).

We next performed live cell imaging on isolated taste receptor cells from PLCβ3-KO and wild type mice to evaluate how the loss of PLCβ3 affects the responsiveness of the BR cells. Type III cells in the PLCβ3-KO mouse were still present and responded to sour and salt stimuli (Fig 5A and 5B). A summary of the response profiles is shown in Fig 5C. Some Type III cells responded to sour (CA, red region) or sour + NaCl (blue region), but none responded to any bitter, sweet, or umami stimuli (yellow region) tested. Type II cells were still functional (Fig 5C, black region) and their response rate was not significantly affected by the loss of PLCβ3 expression (S5 Fig). Example traces of Type II responses are shown in Fig 5D, 5E and 5F. A more comprehensive analysis of the PLCβ3-KO mice found that the loss of PLCβ3 caused a very significant reduction in the number of BR taste cells (Fig 5G) and no BR cells were identified in either the Fol or Fun papillae of the PLCβ3-KO mouse (Fig 5H). While 23.5% of the CV taste cells were BR in the WT mice, only 2% of the CV taste cells were BR in the PLCβ3-KO mouse. Immunohistochemical analysis of the PLCβ3-KO mouse confirmed the absence of any PLCβ3 expression in the taste buds (S6 Fig) compared to WT mice. These data suggest that PLCβ3 is required for transducing most of the bitter, sweet and umami responses in BR cells.

### Loss of PLCβ3 significantly affects taste-driven licking for bitter, sweet and umami

**Fig 6:**
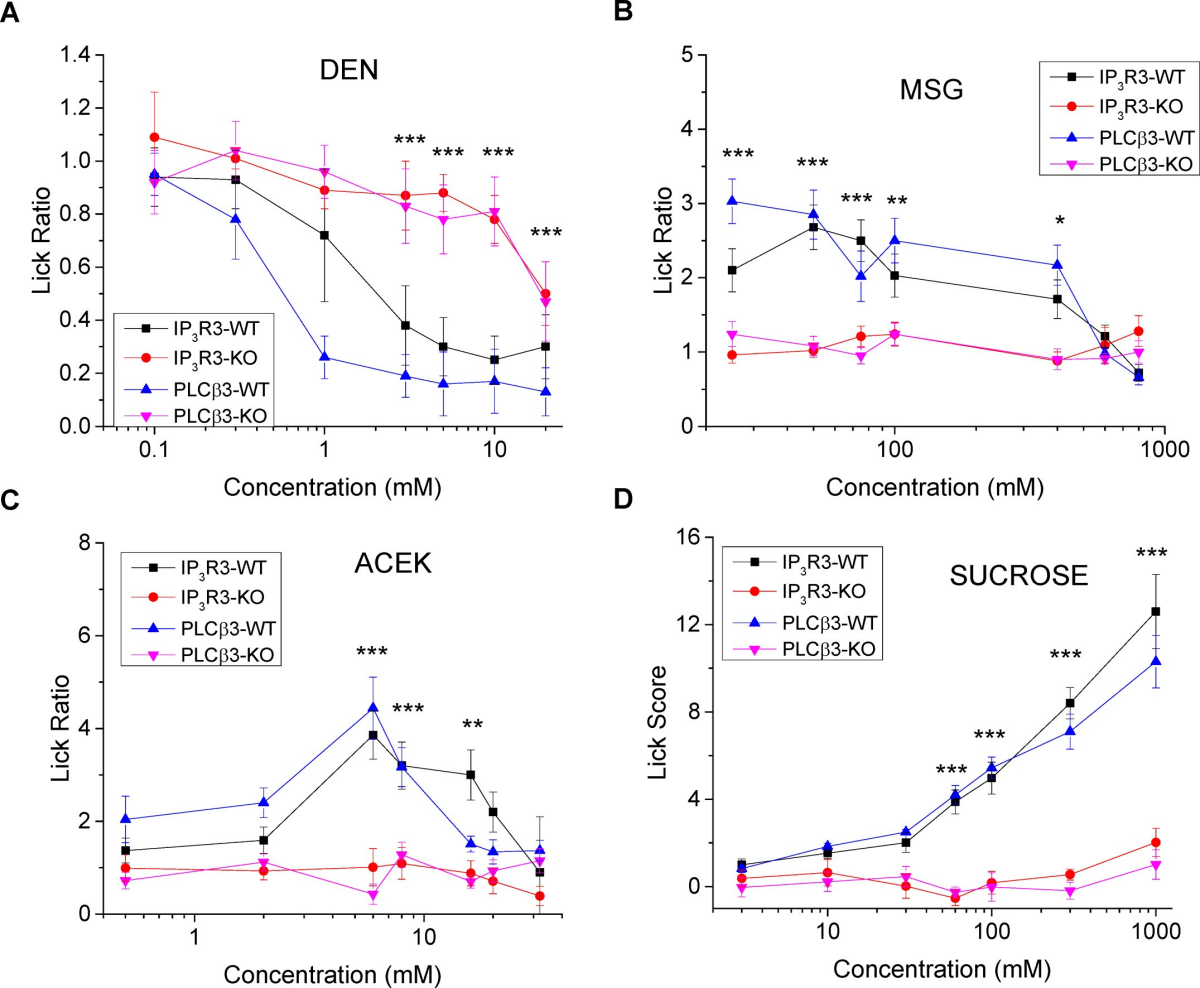
Loss of PLCβ3 or IP_3_R3 affects behavioral responses to taste stimuli. Average lick data (±standard deviation) from brief-access behavioral tests compare the responses of IP_3_R3-KO (red line) and PLCβ3-KO (pink line) to WT (IP_3_R3-WT, black line; PLCβ3-WT, blue line). A) Lick ratios (stimulus/water) of the WT mice for the bitter stimulus denatonium (DEN, 0, 0.1, 0.3, 1, 3, 5, 10, 20mM) were significantly different from the IP_3_R3-KO and PLCβ3-KO responses. B) Lick ratios of the WT mice for the umami stimulus monosodium glutamate + 10μM amiloride (MSG, 0, 25, 50, 75, 100, 400, 600, 800mM) were significantly different from the IP_3_R3-KO and PLCβ3-KO mice. C) Lick ratios of the WT mice for the artificial sweetener acesulfame K (ACEK, 0, 0.5, 2, 6, 8, 16, 20, 32mM) were significantly different from the IP_3_R3-KO and PLCβ3-KO mice. D) Lick scores (stimulus-water) of the WT mice for sucrose (0, 3, 10, 30, 60,100, 300, 1000mM) were significantly different from the IP_3_R3-KO and PLCβ3-KO responses. For all experiments, 5 mice of each genotype were used (***, p<0.001; **, p<0.01; *, p<0.05). Data were compared by repeated measures ANOVA. Significant interaction terms were followed by Tukey’s Honestly Significant Difference tests.

Our data so far demonstrate that a subset of Type III taste cells are capable of responding to bitter, sweet, and umami using a PLCβ3 signaling pathway. To determine if these BR taste cells are important in taste transduction, we performed brief-access licking experiments to determine if the loss of taste-evoked signals in the PLCβ3-KO mice correlated with loss of taste sensitivity at the behavioral level. Measurements were made with IP_3_R3-KO mice for comparison. Since the PLCβ3-KO mouse has a mixed genetic background while the IP_3_R3-KO mice are in a C57BL/6 background, wild type littermates for both mouse lines were used as controls (Fig 6). The wild type mice both demonstrated concentration dependent decreases in licking to denatonium while PLCβ3-KO and IP_3_R3-KO mice treated the solution similarly to water except for the highest concentration (20mM Den, Fig 6A). Wild type mice treated MSG (+ 10μM amiloride) solutions as more palatable than water up to 400mM and then decreased licking at the highest concentrations (600 and 800mM, Fig 6B). Both of the KO mice treated MSG like water. In the brief access test for the artificial sweetener, Acesulfame K (AceK), wild type mice increased licking up to 2 mM and then decreased licking at higher concentrations. Both of the KO mice treated AceK like water (Fig 6C). Because sucrose is hedonically positive under wild type conditions, the lick score is calculated as licks to the stimulus minus licks to water, therefore a score close to zero resembles water. Neither the PLCβ3-KO nor IP_3_R3-KO mice showed a concentration-dependent effect on licking to sucrose until they were presented with the highest concentration. In contrast, both sets of wild type mice showed strong concentration dependent increases in licking to sucrose (Fig 6D). As positive controls, we also tested sodium chloride and citric acid since salt and sour are not predicted to activate a PLCβ signaling pathway in taste cells. We tested NaCl for both IP_3_R3-KO and PLCβ3-KO mice since earlier studies have suggested Type II cells may be involved in salt taste [35]. For the salt stimulus (NaCl), there were no significant differences in the licking behavior for either of the KO mice compared to wild type mice, indicating that the ability to respond to salt is not affected by loss of either PLCβ3 or IP_3_R3 (S7A Fig). We also did not record any differences in the licking responses to sour (citric acid) for the PLCβ3-KO mice compared to WT mice (S7B Fig). Thus, loss of either IP_3_R3 or PLCβ3 caused comparable, and almost complete, loss of behavioral responses specifically for bitter, sweet and umami stimuli.

### Loss of PLCβ3 causes deficits in the taste-evoked activity in the nucleus of the solitary tract

**Fig 7:**
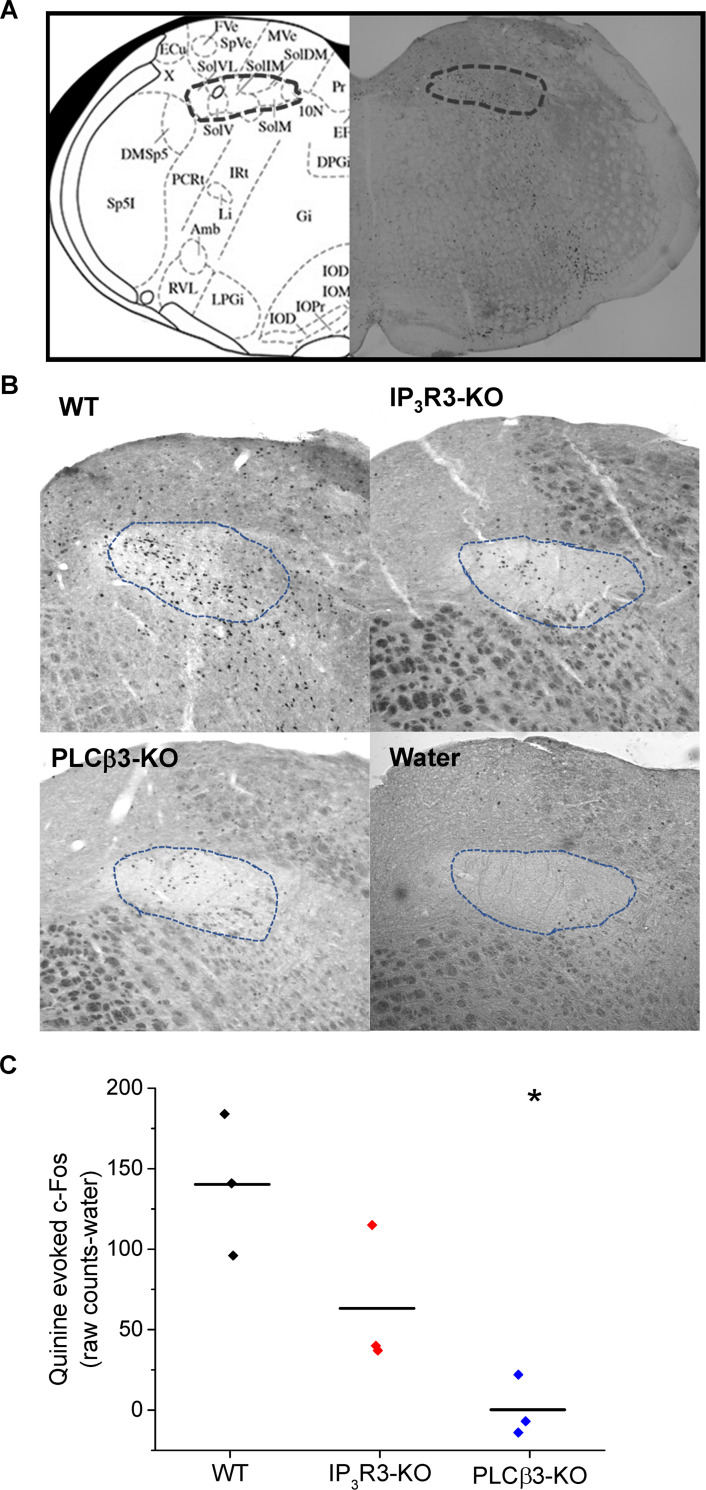
Loss of PLCβ3 or IP_3_R3 causes deficits in the taste-evoked neural activity in the nucleus of solitary tract. Oral infusion of bitter (quinine) and water elicited c-Fos-like immunoreactivity (FLI) in the intermediate rostral nucleus of the solitary tract (IRNTS). A) The schematic diagram indicates the approximate region in the hindbrain that was analyzed. B) The boundaries of the NTS were delineated for each subject and the relative FLI to quinine for each genotype were compared. Loss of either PLCβ3 (PLCβ3-KO) or IP_3_R3 (IP_3_R3-KO) caused a reduction in the FLI in the IRNTS compared to WT mice. C) For all experiments, 3 mice of each genotype were used and data were normalized to the water control. Each data point is plotted and the average values are indicated with a black line for each genotype. Data were analyzed with a one way ANOVA with follow up Fisher’s post-hoc analyses to identify individual differences. Significance level was set at p<0.05 (*).

Although the BR cells respond to bitter, sweet, and umami stimuli in the absence of functional Type II cells, and Type II cells respond normally to these stimuli in the absence of functional BR cells when tested *in vitro*, the loss of either cell type abolishes the behavioral response to bitter, sweet and umami stimuli. To ask if the signal from the taste receptor cells was reaching the brain, where it would be integrated into a behavioral output, we measured the c-fos reactivity in the in the nucleus of the solitary tract (NTS) in the absence of functional BR cells (PLCβ3-KO). The NTS receives input from the gustatory neurons and is the first synaptic relay from the peripheral taste system. This approach is commonly used to identify recently activated neurons, including neurons in the taste pathway [36–40]. We reasoned that if BR cells are contributing to the transduction of taste information to the brain, then the loss of PLCβ3 would have a significant impact on the level of c-Fos labeling in the NTS. We used the IP_3_R3-KO mouse as a positive control since it has previously been shown to be required to transmit bitter, sweet, and umami taste information to the brain [41]. Mice (n = 4 for each mouse line) were orally infused with quinine (5mM) or water for 30 min. Background c-Fos labeling was measured using water as a control. The boundaries of the NTS were delineated individually for each subject and the number of c-Fos positive cells within that area were counted and normalized to water [38]. An overview of the NTS is shown in Fig 7A. As expected, WT mice had a large number of c-Fos positive cells in the NTS (Fig 7B, upper left panel), while the IP_3_R3-KO mice (Fig 7B, upper right panel) and water control mice (Fig 7B, lower right panel) both had few c-Fos positive cells. Strikingly, the level of c-Fos labeling in the PLCβ3-KO mice (Fig 7B, lower left panel) were comparable the c-Fos levels in the IP_3_R3-KO and water control mice. We found that the absence of PLCβ3 resulted in a significant reduction in c-Fos activity in this region of the NTS (Fig 7C). Control experiments demonstrate that neither IP_3_R3 nor PLCβ3 are expressed in this region of the NTS (S8 Fig) so the recorded deficits were not due to the loss of either of these proteins in the NTS.

## Discussion

**Fig 8:**
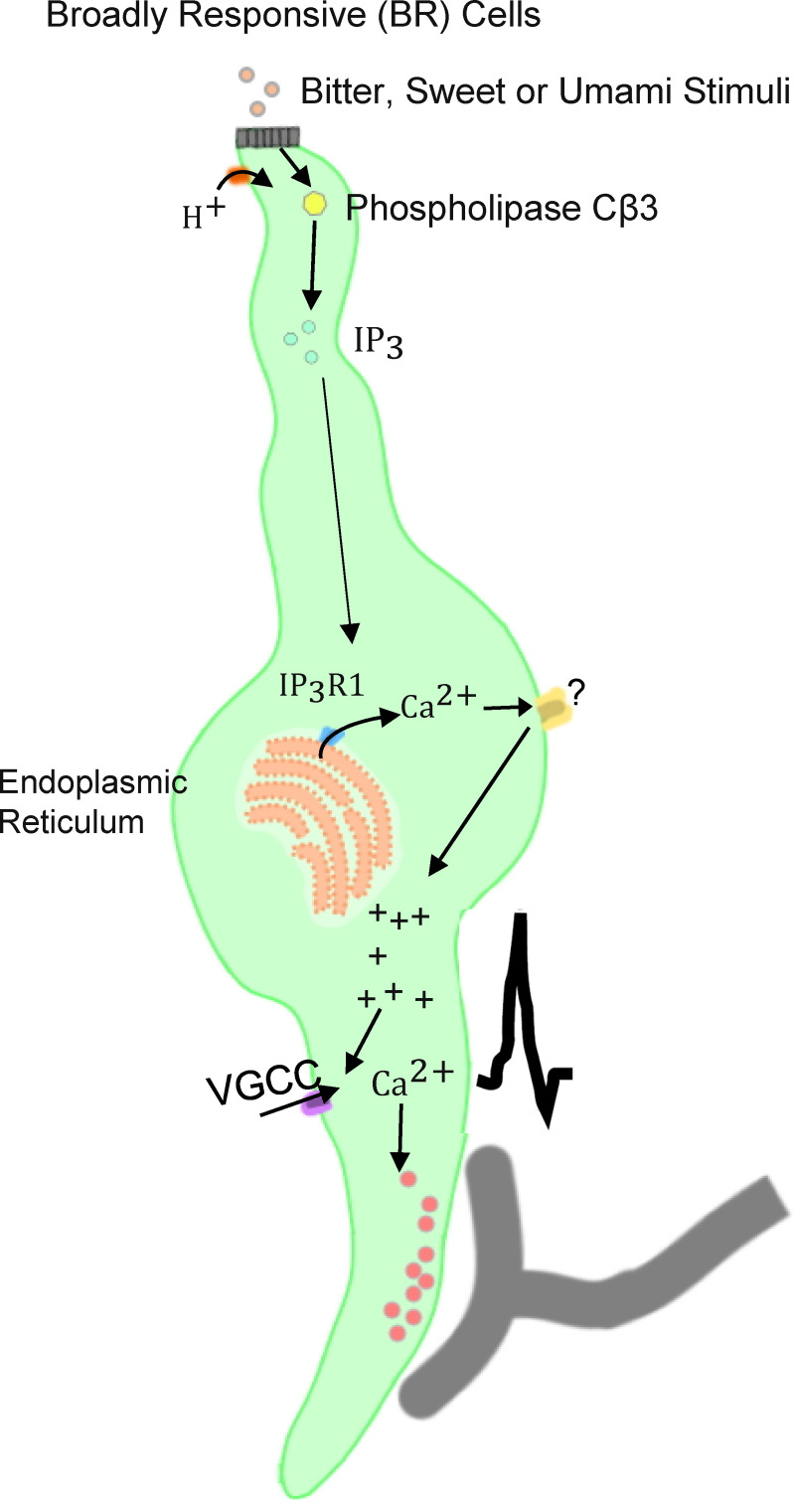
BR taste cell model. A model of the potential signal transduction pathway in BR taste cells. Based on data presented in this study, PLCβ3 is predicted to be required for this pathway to function. We previously identified that PLCβ3 and IP_3_R1 co-localize [23]. Future studies are needed to identify the other proteins in this pathway.

This study has characterized a population of taste cells that express VGCCs, respond to sour stimulation with a Ca^2+^ signal, but are also responsive to bitter, sweet, and/or umami stimuli (See model in Fig 8). Our data suggest that BR cells are a subset of Type III cells that are capable of responding to multiple taste stimuli, except sodium chloride. Since the BR cells always responded to sour, 100% of these cells responded to multiple taste qualities and approximately 80% of these cells responded to either three or four modalities (Fig 3D). This is in contrast to Type II cells that are usually narrowly tuned to a single taste quality. While we are not able to stimulate individual BR cells with all taste stimuli, our data suggest that the BR cells may be very broadly tuned and act as a generalist responding cell. It is unclear why BR cells did not respond to the sodium salt we tested, but other studies have reported that sodium responsive cells appear to be a separate taste cell population [18, 42].

The idea of broadly tuned taste cells in mammals has been put forth by multiple labs, using a variety of approaches that utilized whole taste buds [43–50]. Maintaining the taste cells in the bud allowed for measurements in a more “native” environment and allowed stimuli to be apically applied to the cells. Sato and Beidler [44, 45] inserted a microelectrode into a taste cell through the taste pore, thus leaving the taste bud intact within the tongue of an anesthetized rat. Using this minimally invasive approach, they identified that most taste cells (~85%) responded to more than one type of taste stimuli. Similarly, Gilbertson et al [50] apically applied four basic taste stimuli to taste buds within the epithelium using a modified Ussing chamber. Their approach allowed for the focal application of taste stimuli to the apical end of the taste cells that were still within the bud. Using patch clamp analysis, they found approximately 73% of the cells responded to two or more taste stimuli [50]. Ninomiya’s group analyzed Type II and Type III taste cells in excised mouse taste buds using gustducin-GFP to identify Type II cells and GAD67-GFP to identify Type III cells [31]. They found that Type II taste cells (gustducin-GFP+) were selective in their responses to bitter, sweet, or umami stimuli while all Type III cells (GAD67-GFP+) responded to sour stimuli. Interestingly, they found about 25% of the GAD67-GFP+ cells responded to multiple taste stimuli, including sweet, bitter, and umami in addition to sour [31]. Roper’s lab used calcium imaging in a slice preparation of taste buds in which they focally applied taste stimuli [30, 49] and found that a large percentage of the taste cells were broadly tuned. These studies also concluded that Type II cells are more selective in their responsiveness while Type III cells are sometimes broadly tuned [30].

All of these earlier studies used intact taste buds and in some cases, the authors concluded that the broadly responsive Type III cells were likely receiving input from the neighboring Type II cells [30]. A key difference between our work and these earlier studies is that we used isolated taste cells that were not in contact with neighboring cells. This allowed us to analyze how individual taste cells respond without any potential input from neighboring cells. While we do not rule out the possibility that taste cells receive input from other taste cells, our data show that BR Type III cells do not require neighboring input to respond to bitter, sweet, and/or umami stimuli. Taken with these earlier studies [30, 31, 43–50], our data support the idea that there are both selectively and broadly responsive cells in taste buds. In agreement with others [30, 31], our data indicate that these BR cells are a subset of Type III taste cells and we have now shown that BR cells can independently respond to bitter, sweet, and umami stimuli.

Many studies focused on the upstream components of the taste pathway have also reported that generalist and specialist cell populations exist. Studies of individual afferent gustatory neurons and geniculate ganglion cells found that some gustatory nerves are highly specific to a particular taste stimulus (specialists) while many of these cells are broadly tuned (generalists) [50–56]. At the first synaptic relay of central taste processing, some neurons in the rostral nucleus of the solitary tract (rNTS) respond ‘best’ to a particular taste stimulus, while others are broadly tuned [57, 58]. Specialists and generalists cells have also been described in the gustatory cortex [59–63]. Our data demonstrate that taste receptor cells, the first step in the taste pathway, mirror the functional heterogeneity that is found higher up in the taste processing; some taste cells are selective (Type II taste cells) to a particular stimulus, while others are broadly tuned (BR taste cells) to multiple taste stimuli.

While we previously reported that PLCβ3 is expressed in a subset of taste cells that are not Type II cells [23], we have now demonstrated PLCβ3 is present in a subset of Type III cells. Data from the PLCβ3-KO mouse (Fig 5) strongly suggests that PLCβ3 is necessary for BR cells to respond to bitter, sweet, and/or umami stimuli. This idea is supported by recent RNA-sequencing analysis of individual Type II and Type III cells [27] which identifies PLCβ3 expression in 25% of Type III cells. We searched this RNA-seq dataset of Type III cells [27] (normalized counts in Table S3, threshold >50) to determine if other signaling components associated with PLCβ signaling are expressed in Type III taste cells. We found that the alpha G-protein Gαq which activates PLCβ, is expressed in these cells. We also identified IP_3_R1 expression in Type III cells, which is a downstream target of PLC [27]. In Type II cells, PLCβ2 activates IP_3_R3 which causes a Ca^2+^ release to activate TRPM4 and TRPM5 (see Fig 1). Our search of the RNA-seq dataset did not find TRPM5 in Type III cells, but did identify TRPM4 expression in all of the Type III cells that expressed PLCβ3, suggesting a possible downstream target of the Ca^2+^ release signal in the BR cells [27]. Future studies are needed to determine if these proteins function together in BR cells.

These data are surprising because it is currently thought that Type III cells primarily respond to sour or salt stimuli. Several studies focused on understanding Type III taste cells targeted and ablated PKD2L1 expressing cells since PKD2L1 is thought to be expressed in all Type III taste cells. They found that loss of these cells abolished the gustatory nerve responses to sour but did not alter gustatory nerve signaling to bitter, sweet, and umami [22, 64]. While clearly Type II cells were still functional in those studies, our data suggest the BR cells are also needed for normal bitter, sweet, and umami gustatory processing. A separate report found that Type III cells are heterogeneous and do not uniformly express the same cell markers [32], so it may be that the BR cells are separate from the PKD2L1 expressing Type III cells. Recently, OTOP1, which is expressed in Type III taste cells, has been proposed as the sour receptor [56, 65, 66]. Nerve recordings from OTOP1-KO mice were significantly reduced for sour stimuli but also showed some reduction in their responsiveness to bitter, sweet, and umami [66]. Taken together, these data highlight a heterogeneity in the characteristics of Type III taste cells that has not yet been well defined.

After taste receptors for bitter, sweet, and umami were molecularly identified [67–71], multiple labs focused on identifying where these receptors are expressed in taste cells. While most taste receptors (T1Rs and T2Rs) are thought to be expressed only in Type II taste cells [4], there is some heterogeneity in their expression [72–76] and one RNA-seq dataset reported very low levels of expression for both T1R1 and T1R3 in some Type III cells [27]. This study evaluated 14 identified Type III cells, so it is possible that a larger analysis would identify more Type III cells that express these taste GPCRs [27]. In addition to these studies, multiple laboratories have independently concluded that the identified taste receptors are not solely responsible for transducing all bitter, sweet, and umami stimuli. For instance, the detection of some carbohydrates is not impaired by the loss of T1R2 or T1R3, the identified sweet receptors [77–82], and umami stimuli appear to be transduced by receptors in addition to the T1R1+T1R3 heterodimer [83–91]. The glucose transporter which has been implicated in detecting sweet stimuli [92], was also expressed in some Type III cells in the RNA-seq profiling study. Our findings support the idea that there may be additional taste receptor mechanisms, outside of those canonically identified, involved in the detection of bitter, sweet, and umami stimuli. While our study is not focused on identifying the receptors that activate the PLCβ3 signaling pathway, our data indicate that future studies are needed to identify which taste receptors are important in BR cells.

Even though the BR cells are only a subset of cells within the bud, our data suggest that these cells make a significant contribution to taste. The loss of PLCβ3 caused very significant impairments in the behavioral responses to bitter, sweet, and umami stimuli (Fig 6). These deficits were comparable to the loss of IP_3_R3 in the Type II cells which has a well-established role in the transduction of bitter, sweet, and umami stimuli. The effects of losing either protein were specific to these taste stimuli and did not affect salt or sour taste (S7A and S7B Fig). As an extra level of support for our hypothesis, we also measured the neural activity in the NTS using c-Fos labeling as a marker for activated neurons to ask if the loss of PLCβ3 affected central processing of taste information from the periphery (Fig 7). We used oral infusions of taste stimuli in awake and behaving animals because it removes any potential confounds due to anesthetization that is used in other approaches. Since the NTS receives input from the gustatory neurons and is the first synaptic relay from the peripheral taste system, these data suggest that loss of PLCβ3 in the taste cells significantly reduced the taste signal that is sent to the brain.

Since PLCβ3 and IP_3_R3 are not expressed in the NTS (S8 Fig) or the geniculate ganglia [93], our data suggest that input from both of these signaling molecules in taste cells is required for normal taste behavior. Moreover, if Type II and BR taste cells independently signal to the central taste system, then the loss of input from either cell population would be predicted to reduce the NTS and behavioral activity proportionally to their contribution in taste processing. However, our data do not support this prediction since the loss of either PLCβ3 (in BR cells) or IP_3_R3 (in Type II cells) significantly impaired both behavioral responses and NTS activity. This suggests that input from both Type II (IP_3_R3 expressing) *and* BR (PLCβ3 expressing) cells may be required for an optimal taste response. When either cell population is non-functional, there are severe deficits in behavioral responses as well as a reduction in the c-fos activity in the NTS.

To date, it is unclear where the information from these two cell groups integrates. It is possible these cells communicate with each other in the bud and that the initial output signal is from one population of taste cells that is receiving input from the other cell group. It is also possible that these two cell populations are independently signaling to the gustatory nerve endings that require input from both cell groups (Type II and BR cells) in order to relay that information to the brain. Based on our data, input from both groups of cells is needed to activate the neurons in the NTS, at least for quinine. Future work is needed to define this relationship for other taste qualities and to examine changes in cell activity in the subnuclei across the rostral NTS. However, our data suggests that the initial stimulus information is integrated relatively early in the gustatory circuit before signal processing occurs in the brain. Regardless of the mechanism, our data suggests that both cell groups need to be functioning to generate appropriate behavioral responses. This type of synergistic relationship is present in multiple sensory systems [94–100] but to our knowledge, it has not previously been described in the taste system. Future studies are needed to better understand this potential relationship.

The presence of broadly tuned taste cells raises an interesting question about how taste information is coded to the brain. One school of thought is that taste information is carried in a labeled line; individual taste cells respond to a particular taste stimulus and this specificity is maintained throughout the taste system [101]. If this is the case, then it is unclear what role BR cells have in coding taste information. Conversely, another school of thought is that taste information is coded using an across fiber approach. Gilbertson et al [50] suggested that multicomponent messages have a greater capacity to transmit information. Encoding information with patterns of activity across broadly tuned cells allows for the transmission of more information than a system using specifically tuned cells [50, 102]. Having broadly tuned cells would allow the taste system to better discriminate between chemicals with similar characteristics. The presence of specialist and generalist cell populations within the taste system suggests that taste coding may be incorporating aspects of both labeled line and across fiber coding. Future studies are needed to address this possibility.

Our study has demonstrated that a subset of Type III cells responds to bitter, sweet, and/or umami stimuli using a PLCβ3 signaling pathway. These cells are broadly tuned to multiple stimuli, including stimuli from distinct taste modalities. This subset of Type III cells appears to have an important role in taste since the loss of either IP_3_R3 (in Type II cells) or PLCβ3 (in BR cells) causes similar deficits in both NTS responses and taste driven behaviors. We conclude that peripheral taste transduction is more complex than is currently appreciated.

## Materials and methods

### Mice

All animal studies were approved by the University at Buffalo Animal Care and Use Committee under protocol number #BIO010174N following the AAALAC guidelines (accreditation # 000039). Multiple mouse lines were used in this study. We did not find any differences in the taste responses between male and female mice in our studies and both sexes were used for all experiments, in approximately equal numbers. Mice ranged in age from 1 to 6 months. Taste cells for qPCR analyses were collected from C57BL/6 mice. The IP_3_R3-KO mouse was generated in a C57BL/6 background and was obtained from the Mutant Mouse Resources and Research Center (MMRRC:032884-JAX). This mouse has a targeted mutation in which exon 1 is replaced with a MAPT/GFP fusion [25] which results in the expression of GFP in place of a functional IP_3_R3 receptor. Thus, these mice lack a functional IP_3_R3 receptor and express GFP in cells that would otherwise have expressed IP_3_R3. Heterozygous mice express both GFP and the IP_3_R3 receptor. The PLCβ3-KO mouse was generously provided by Dr. Sang-Kyou Han [103]. The mutation in these mice disrupts the catalytic domain of phospholipase C and was generated in a 129SV agouti mouse strain that was crossed with CD1 mice [104]. These mice were maintained in this mixed background. Immunohistochemical analyses were also performed in the TRPM5-GFP mouse strain which expresses GFP in all taste cells that express TRPM5. These mice have been backcrossed into the C57BL/6 background and were used as another marker of Type II taste cells [15] to evaluate the expression patterns of PLCβ3. These mice were generously provided by Dr. Robert Margolskee. The GAD67-GFP mice were purchased from the Jackson Laboratory (Cat#007677).

### Taste receptor cell isolation

Taste receptor cells were harvested from CV, Fol and Fun papillae of adult mice as previously described [23, 105–110]. Briefly, mice were sacrificed using carbon dioxide and cervical dislocation. Tongues were removed and an enzyme solution containing 0.7mg/mL Collagenase B (Roche, Basel, Switzerland), 3mg/mL Dispase II (Roche), and 1mg/mL Trypsin Inhibitor (Sigma-Aldrich, St. Louis, MO) was injected beneath the lingual epithelium. After the tongues were incubated in oxygenated Tyrode’s solution for approximately 17 min, the epithelium was peeled from the underlying muscle and pinned serosal side up before it was incubated in Ca^2+^-free Tyrode’s for approximately 25 min. Cells were removed from taste papillae using capillary pipettes with gentle suction and placed onto coverslips coated with Cell-Tak (Corning, Corning, NY).

### Ca^2+^ imaging

All measurements of intracellular calcium (Ca^2+^) were performed in isolated taste receptor cells that were no longer in contact with other taste cells. Cells were loaded for 20 minutes at room temperature (RT) with 2 μM Fura2-AM (Molecular Probes, Invitrogen, Carlsbad, CA) containing 0.05% Pluronic F-127 (Molecular Probes). Loaded cells were then washed in Tyrode’s solution under constant perfusion for 20min. Multiple taste stimuli and high potassium (50mM KCl) solutions were individually applied and Ca^2+^ responses were recorded. Cells were visualized using an Olympus IX73 microscope with a 40x oil immersion lens and images were captured with a Hamamatsu ORCA-03G camera (Hamamatsu Photonics K.K., SZK Japan). Excitation wavelengths of 340nm and 380nm were used with an emission wavelength of 510nm. Cells were kept under constant perfusion using a gravity flow perfusion system (Automate Scientific, San Francisco, CA). Images were collected every 4s using Imaging Workbench 6.0 (Indec Biosystems, Santa Clara, CA). Experiments were graphed and analyzed using Origin 9.2 software (OriginLab, Northhampton, MA).

Intracellular Ca^2+^ levels were measured as a ratio of fluorescence intensities. Fluorescence values were calibrated using the Fura-2 Ca^2+^ Imaging Calibration kit (Invitrogen). The effective dissociation constant K_d_ was calculated to be 180nM, which was used in the following equation to calculate Ca^2+^ concentration:

$$\left[Ca^{2+}\right]=K_{d}\left[\left(R‐R_{min}\right)/\left(R_{max}‐R\right)\right]\left(S_{f2}/S_{b2}\right)$$

$$\left[Ca^{2+}\right]=0.180\;\left[\left(R‐0.338\right)/\left(26.81‐R\right)\right]\left(22.19\right)$$

R is the ratio value of fluorescence obtained after exciting cells at 340 and 380nm. Data from cells were analyzed if the cell had a stable Ca^2+^ baseline within the range of 65nM and 200nM. An evoked response was defined as measurable if the increase in fluorescence was at least two standard deviations above baseline.

### Immunohistochemistry

Mice were deeply anesthetized by intraperitoneal injections of sodium pentobarbitol, 40 mg/kg (Patterson Veterinary, Mason, MI). Mice were then injected intracardially with heparin (Sigma) and 1% sodium nitrite (Sigma) followed by perfusion with approximately 30mL of 4% paraformaldehyde (Electron Microscopy Sciences, Ft. Washington, PA) in 0.1M phosphate buffer (PB), pH 7.2. After perfusion, the tongues were removed and placed in 4% paraformaldehyde/0.1M PB for 1-2h followed by a 4°C overnight incubation in 20% sucrose/0.1M PB, pH 7.2. For some experiments, tongues were immersion fixed overnight in 4% paraformaldehyde/0.1M PB, pH 7.2 at 4°C with 20% sucrose. Regardless of the fixation method, the next day, 40μm sections were cut and washed in PBS 3X10 min at RT. For some experiments, antigen retrieval was performed by placing sections in 10 mM sodium citrate, pH 8.5 at 80°C for 5 min. This was done to disrupt the cross-bridges formed by fixation and expose antigen binding sites.

Sections were incubated in blocking solution (0.3% Triton X-100, 1% normal goat serum and 1% bovine serum albumin in 0.1M PB) for 1-2h at RT. Primary antibody was added to the sections in blocking solution and incubated for 2 hours at RT followed by overnight exposure to primary antibody at 4°C. Controls with no primary antibody were included in each experiment. All primary antibodies were diluted in blocking solution. Mouse anti-IP_3_R3 (BD Biosciences; San Jose, CA, Cat#. 610313; RRID: AB-397705) was used at 1:50 following antigen retrieval. Rabbit anti-PLCβ3 (Abcam, Cambridge, MA, Cat# ab52199, RRID:AB-2236995) was used at 1:200, rabbit anti-PLCβ2 (Santa Cruz Laboratories, Santa Cruz, CA, Cat# SC-206, RRID: AB-632197) was used at 1:1000, rabbit anti-gustducin (Santa Cruz, Cat# SC-395, RRID: AB-10177605) was used at 1:200 and anti-NTPDase2 was used at 1:100 (J. Sevigny, Laval University; Quebec; Canada Cat# NTPDase2, RRID:AB_2314986) [111]. Mouse anti-SNAP-25 (Genway Biotech, San Diego, CA, Cat# 20-783-70323, RRID: AB-1024914) was used at 1:200 following antigen retrieval. Following overnight incubation in primary antibody, sections were washed in PBS 3X10 min at RT and then incubated with the appropriate secondary antibody (Cy5, 1:500; Rhod, 1:250; Jackson ImmunoResearch Laboratories, West Grove, PA) at RT for 2h in the dark. Controls were performed for double labeling experiments to ensure secondary antibodies were not binding to primary antibodies raised in different organisms. After secondary antibody incubation, sections were washed in PBS (3x10 min) and mounted on Superfrost Plus slides (VWR, Radnor, PA) using Fluoromount G (Southern Biotechnology Associates, Birmingham, AL) and coverslipped. All images were obtained using a Zeiss LSM 710 Confocal Microscope (Zeiss, Oberkochen, Germany). Stacks were collected using Zen software (Zeiss) and images were processed using Adobe Photoshop CS5 software adjusting only brightness and contrast.

### Real-time PCR of isolated taste cells

Taste receptor cells from CV, Fol or Fun papillae were isolated from the papillae as described above and then centrifuged for 20min at 13,000 RPM. RNA was purified using the NucleoSpin RNA XS Kit (Macherey-Nagel, Düren, Germany) according to kit instructions. PCR analysis was performed for GAPDH to ensure sample quality and check for genomic contamination. Contaminated samples were discarded and new samples were collected. Real-Time PCR was performed using a BioRad MiniOpticon system (Bio-Rad Laboratories, Hercules, CA), with BioRad SYBR Green reagents (Bio-Rad Laboratories). Primers used for these experiments were: **PLC**β**2**, *Fwd*: CAATTGAGGGGCAGCTGAGA *Rev*: TTCTAGGCTGCATCTGGGC; **PLC**β**3**, *Fwd*: TCCTGGTGGTCAGGGAT *Rev*: CTGCCTGTCTCTGCTATCCG; **GAPDH** *Fwd*: ACAGTCAGCCGCATCTTCTT, *Rev*: ACGACCAAATCCGTTGACTC.

For real-time PCR analyses, each sample was run in triplicate. If there was more than 5% difference between the replicates, the data were discarded. Multiple biological repeats were used for each papillae type (CV, n = 6; Fol, n = 6; Fun, n = 5). Data was normalized to GAPDH expression for each sample to correct for any loading differences and reported as fold differences.

### Analysis of licking behavior

Unconditioned licking responses to varying concentrations of taste stimuli were recorded in a test chamber designed to measure brief-access licking (Davis MS80 Rig; Dilog Instruments and Systems, Tallahassee, FL). This apparatus consisted of a Plexiglas cage with a wire mesh floor. An opening at the front of the cage allowed access to one of sixteen spill-proof glass drinking tubes that reside on a sliding platform. A mechanical shutter opened and closed to allow the mouse access to one of the tubes for a user-specified length of time. A computer controlled the movement of the platform, order of tube presentation, opening and closing of the shutter, duration of tube access and interval between tube presentations. Each individual lick was detected by a contact lickometer and recorded on a computer via DavisPro collection software (Dilog Instruments and Systems).

Mice were adapted to the test chamber and trained to drink from the sipper tubes for 5 consecutive days as previously described [112, 113]. During training, mice were 20-h water deprived. On the first day of training, the mouse was presented with a single stationary bottle of water for 30 min. On the second day, a tube containing water was presented but this time the mouse was given 180s to initiate licking and once licking was recorded the mouse was given 30s access to the tube. At the conclusion of either the 30s access or the 180s limit, the shutter was closed again for 10s. Each of the 8 tubes, all containing water, was presented 3 times. During the remaining three days of training, the mouse was given 30 min to initiate licking to one of eight tubes of water. Once the mouse began licking, it was given 10s to lick before the shutter closed for 10s, after which a new tube was presented.

During testing, animals were allowed to take as many trials as possible in 30 min. Mice were tested on varying concentrations of sucrose (0,3,10,30,60,100,300,1000 mM), acesulfame K (0,0.5,2,6,8,16,20,32 mM), MSG with 10 μM amiloride (0,25,50,75,100,400,600,800 mM), denatonium benzoate (0,0.1,0.3,1,3,5,10,20 mM), and NaCl (0,3,10,30,60,100,300,1000 mM), in that order. Citric acid (0, 1, 3, 10, 30, 100 mM) was presented to PLCβ3 WT and KO mice. Each stimulus was presented in randomized blocks on Monday, Wednesday and Friday in a single week. Animals were 22-h water deprived for all testing except sucrose, when animals were tested water replete. Once the animal began licking the tube, they were allowed 10 seconds of access before the shutter closed.

For stimuli tested in the water deprived condition (acesulfame K, MSG + amiloride, denatonium benzoate, NaCl, and CA), lick ratios were calculated by dividing the average number of licks at each concentration by the average number of licks to water. For stimuli tested while the animals were water replete (sucrose), licks scores were calculated by subtracting the average number of licks at each concentration by the average number of licks to water. These corrections were used to standardize for individual differences in lick rate and were based on water need. Lick scores and licks relative to water were compared by repeated measures ANOVA with genotype as between factors variable and concentration as a repeated measures within factors variable. Significant interaction terms were followed by Tukey’s Honestly Significant Difference tests. Statistical analyses were performed in Statistica.

### Oral infusions

Wild type, IP_3_R3-KO and PLCβ3-KO mice (n = 3 for each) were tested to measure the effects of quinine stimulation on the c-Fos immunoreactivity in the intermediate rostral nucleus of the solitary tract (NTS). Water infusions (n = 3) were performed to measure background responses. Surgical procedures were conducted using sterile technique while mice were anesthetized via 2% - 4% isoflurane anesthesia. Guided by a 21G needle, a small length of PE-90 tubing was passed through the animal’s cheek, emerging into the oral cavity by the rear molars. The tubing was threaded through Teflon washers and heat flared to secure on both the external and oral sides. Mice were given an analgesic (0.5mg/ml of carprofen) and allowed to recover. After recovery, they were infused with either quinine (5mM) or water into the oral catheter for 30 minutes (0.2ml/min, 30 min infusion). Following the infusion, animals were returned to their cages and left undisturbed for 45 minutes.

### Brain histology and analysis

After the 45 minute post-infusion period, mice were perfused as described above and the hindbrains were removed. The following day, hindbrains were sectioned into 40μm coronal sections which were washed in TBS (pH 7.5), 3x10 min each. Sections were incubated in 1:100 H_2_O_2_ in TBS for 15 min, followed by 3X10 min TBS washes. Sections were then incubated in anti-c-Fos (Abcam Cat# ab190289, RRID:AB_2737414, 1:1000) diluted in blocking buffer (3% Normal Donkey Serum in TBS-TritonX) for 1 hr at RT. This was followed by an overnight incubation at 4°C. The next day, sections were washed 3x10 min in TBS and then incubated for 2 hrs in anti-rabbit secondary antibody (711-065-152, Jackson Immunoresearch, 1:1000) in blocking buffer. After incubation, sections were kept in an avidin-biotin mixture (Elite kit; Vector Laboratories) in TBS-TritonX for 1 hr. Tissue sections were washed (3x10 min in TBS) and then stained with DAB (3,3’-diaminobenzidine-HCL; Vector Laboratories) for 5 min. Stained tissue sections were washed (3x5 min in TBS) and mounted onto slides using Fluoromount-G (Southern Biotechnology Associates). Sections were examined using a light microscope (10–100 X) equipped with a digital camera.

Analyses of digital images were performed on the intermediate rostral nucleus of the solitary tract (iRNTS; ~500 μm caudal to the dorsal cochlear nucleus) which receives afferents from both the glossopharyngeal and chorda tympani nerves and displays dense c-Fos-like immunoreactivity (FLI) in response to intraoral delivery of quinine [37, 38, 114]. The boundaries of the iRNTS were delineated based on previously published reports [37, 38, 114] and used the distance from the fourth ventricle and vestibular nuclei as anatomical landmarks. These boundaries were drawn for each individual subject and the number of FLI-positive neurons contained within the area was counted by hand by an experimenter blind to genotype. We measured the area of the nucleus that was analyzed in each subject as a method of demonstrating that differences in FLI presence were not due to inherent differences in the size of the nucleus. These areas were compared with a one-way ANOVA which revealed no difference in area as a function of genotype (F_(2,9)_ = 1.63, *P* = 0.24). As we were solely interested in taste-evoked FLI, the number of FLI generated by intraoral water infusions for each genotype was subtracted from the quinine-evoked FLI and these counts were compared. Finally, in order to increase our confidence in the reliability of the identification of FLI-positive neurons within the NTS, a second blind experimenter counted the sections. The inter-rater reliability correlation between these counts was 0.94 indicating high correlation in the identification and sum of FLI-positive neurons in the iRNTS. Data were analyzed with a one way ANOVA with Fisher’s post-hoc analyses to identify individual differences. Significance level was set at p<0.05.

### Solutions

All chemicals were purchased from Sigma Chemical (Sigma-Aldrich, St. Louis, MO) unless otherwise noted. Tyrode's solution contained 140mM NaCl, 5mM KCl, 3mM CaCl_2_, 1mM MgCl_2_, 10mM HEPES, 10mM glucose, and 1mM pyruvate, pH 7.4. Ca^2+^-free Tyrode's contained 140mM NaCl, 5mM KCl, 2.7mM BAPTA, 2mM EGTA, 10mM HEPES, 10mM glucose, 1mM pyruvate, pH 7.4. Nominal Ca^2+-^free Tyrode's contained 140mM NaCl, 5mM KCl, 10mM HEPES, 10mM glucose, 1mM pyruvate, pH 7.4. Hi KCl solution contained 50mM KCl, 90mM NaCl, 3mM CaCl_2_, 1mM MgCl_2,_ 10mM HEPES, 10mM glucose, 1mM pyruvate, pH 7.4. All taste-solutions, 2μM U73122 (Tocris, Bristol, United Kingdom), and 2μM thapsigargin (Tocris) were prepared in normal Tyrode's.

In live cell imaging, multiple taste stimuli were analyzed for bitter (5mM denatonium benzoate (Den)), sweet (20mM sucralose, 2mM saccharin, 50mM sucrose, 20mM Acesulfame K (Ace K)), and umami (10mM monopotassium glutamate (MPG)). 50mM sucrose contained 90mM NaCl instead of 140mM. Salt (250mM sodium chloride, NaCl) and sour (50mM citric acid, CA, pH4) stimuli were tested using the same protocol as Lewandowski et al [18].

### Statistics

Analysis of the taste responsiveness (response rate) was performed using an interactive Chi-square analysis with Yate’s correction for continuity [115]. Significant differences were reported if p<0.05. For real-time PCR analyses, a one-way ANOVA with p<0.05 set as the limit of significance was used to identify any significant differences between the relative expression of PLCβ2 and PLCβ3 in the different papillae types. Comparisons between two experimental conditions were made using a two-tailed Student’s T test with p<0.05 set as the limit of significance.

### Colocalization analysis

Image stacks (n = 5, 1 μm each) were acquired from labelled sections obtained from three different animals and regions of interest (ROI) were drawn to include the area inside clearly identifiable taste buds. Colocalization analysis was performed on the ROIs using ImageJ Fiji software (NIH). Colocalization was determined based on Pearson’s coefficient using the Colocalization_Finder plugin. If the Pearson’s coefficient value was greater than 0.9, we considered it to be 100% colocalization due to the variability in immunofluorescence intensity. If the Pearson’s coefficient was less than 0.05, we considered this to be no colocalization. A Pearson’s coefficient higher than 0.05 but lower than 0.9 was considered as partial colocalization.

## Supporting information

S1 Fig Characterization of the IP_3_R3-KO mice. A) Laser scanning confocal micrographs (LSCMs, stack of 5 slices, 1μm each) from WT mice identified anti-IP_3_R3 labeling in taste receptor cells from the CV (n = 3). B) IP_3_R3-KO mice express GFP in lieu of IP_3_R3 and were not labeled by anti-IP_3_R3 (n = 6). C) LSCMs of the IP_3_R3-het mouse identified strong co-localization between anti-IP_3_R3 labeling and GFP expression. D) Anti-PLCβ2 labeling in IP_3_R3-KO mice found that PLCβ2 co-localizes with GFP, indicating that IP_3_R3-KO-GFP is specific to Type II cells (LSCMs: stack of 5 slices, 1μm each; n = 4). E) α-gustducin is present in a subset of the IP_3_R3-KO-GFP taste cells in the CV (LSCMs: stack of 10 slices, 1μm each; n = 3). Asterisks identify some GFP expressing cells that do not express gustducin. Scale bars = 20 μm. F) Co-localization analysis identified the average (± standard deviation) overlapping expression for each target protein with the GFP expression, n = 3 for each.
(TIF)

S2 Fig Dual responsive cells are present in multiple mouse lines. Control imaging experiments were performed using GAD67-GFP mice and C57BL/6 mice. GAD67-GFP is expressed in a large subset of Type III mouse taste cells [28]. A-B) Representative traces of BR taste cells that responded to bitter (denatonium = Den), sweet (sucralose = Sucr) and/or umami stimuli (MPG) and 50mM KCl (Hi K) in GAD67-GFP mice. BR taste cells were present in both GAD67-GFP + (A) and GFP- (B) taste cells. C) Experiments in C57BL/6 mice also identified the presence of BR taste cells.
(TIFF)

S3 Fig Taste-evoked Ca^2+^ release in IP_3_R3-KO mice is dependent upon PLC activity and Ca^2+^ release from internal stores. Representative data related to Fig 4. Open columns represent the time that the taste stimulus is presented (40s). The application of Ca^2+^ free Tyrode’s is indicated by the dashed lines. The stimulus presented during this time is also in Ca^2+^ free Tyrode’s. The gray hatched columns represent the application of either thapsigargin (Thap) or U73122, both of which are irreversible inhibitors. A) Bitter-evoked taste responses (5mM Den) persist in the absence of extracellular calcium (Ca^2+^-free) and are abolished by the SERCA pump inhibitor thapsigargin (B) as well as the PLC blocker U73122 (C). D) Responses to sweet stimuli (20mM sucralose, Sucr) persist in Ca^2+^-free and are abolished by thapsigargin (E) and U73122 (F). G) Umami stimuli (10mM MPG) persist in Ca^2+^-free and were abolished by thapsigargin (H) and U73122 (I).
(TIFF)

S4 Fig Expression of PLCβ3 in taste cells. A) Laser scanning confocal micrographs (LSCMs, stack of 5 slices, 1μm each) of PLC**β**3 immunostaining in the IP_3_R3-KO-GFP mice reveal that PLC**β**3 is expressed in a separate population from the GFP positive taste cells in the CV. B) Anti-PLC**β**3 labeling in the CV of TRPM5-GFP mice determined that PLC**β**3 is expressed in taste cells lacking GFP expression (LSCMs: stack of 5 slices, 1μm each; n = 4). C) Co-labeling with anti-PLC**β**2 and anti-PLC**β**3 in the CV of C57BL/6 mice revealed that these PLC**β**s are expressed in separate taste cell populations (LSCMs: stack of 5 slices, 1μm each; n = 3). D) Co-labeling with anti-NTDPase2 and anti-PLC**β**3 in the CV of C57BL/6 mice determined that these markers are expressed in separate taste cell populations (LSCMs: stack of 5 slices, 1**β**m each; n = 3). Scale bar = 20μm. E) Anti-PLC**β**3 labeling in the GAD67-GFP mice determined that PLC**β**3 is partially expressed in taste cells with GFP expression (LSCMs: stack of 5 slices, 1μm each; n = 4). F) Immunohistochemical analyses (LSCMs: stack of 5 slices, 1μm each) using anti-PLC**β**3 and anti-SNAP25 revealed some co-localization between PLC**β**3 and SNAP25 in CV papillae. Scale bars = 10μm. G) Co-localization analysis identified the average (± standard deviation) overlapping expression for PLC**β**3 with TRPM5-GFP, anti-PLC**β**2, IP_3_R3-GFP, or anti-SNAP25 expression, n = 3 for each. mRNA was isolated from taste cells originating in the different papillae types from C57BL/6 mice. Taste cells were analyzed from at least five different mice for each. Values were normalized to GAPDH expression and are presented as a ratio to values from the CV papillae for (H) PLC**β**2 and (I) PLC**β**3. (***, p<0.001).
(TIFF)

S5 Fig Loss of PLCβ3 expression does not affect Type II TRC responses. Chi square analysis with Yate’s correction for continuity was used to compare the response rate or frequency of evoked Ca^2+^ responses to different taste stimuli between wild type (black bars) and PLC**β**3-KO (red bars) mice for taste cells from CV (A), Fol (B), and Fun (C) papillae. D). Table of the stimulus response rate for each papillae type in WT and KO mice. P values for each comparison are also shown. No significant differences were found for any of the comparisons.
(TIFF)

S6 Fig Characterization of the PLCβ3-KO mice. A) LSCMs (stack of 5 slices, 1μm each) from WT mice identified anti-PLC**β**3 labeling in taste receptor cells from the CV (n = 3). B) CV taste cells from the PLC**β**3-KO mice were not labeled by anti-PLC**β**3 (n = 3). Scale bar = 10μm.
(TIF)

S7 Fig Loss of PLCβ3 or IP_3_R3 does not affect behavioral responses to salt or sour stimuli. Average lick data (±standard deviation) from brief-access behavioral tests compare the responses of IP_3_R3-KO (red line) and PLC**β**3-KO (pink line) to WT (IP_3_R3-WT, black line; PLC**β**3-WT, blue line). A) No significant differences were detected between the WT and KO mice for any concentration of NaCl (0, 3, 10, 30, 60, 100, 300, 1000mM) tested. B) No significant differences were detected between the PLC**β**3 WT (blue line) and KO (pink line) mice for any concentration of citric acid (0, 1, 3, 10, 30, 100mM) tested. For all experiments, 5 mice of each genotype were used. Data were compared by repeated measures ANOVA. Significant interaction terms were followed by Tukey’s Honestly Significant Difference tests.
(TIF)

S8 Fig Neither IP_3_R3 nor PLCβ3 is expressed in IRNTS. CV papillae and IRNTS sections were evaluated in parallel. A) Analyses of the brain sections from the IP_3_R3-KO mice revealed no GFP labelling in the IRNTS. Immunohistochemical analysis using anti-PLC**β**3 also did not detect any PLC**β**3 expression. Scale bar = 50μm. B) LSCM analyses (n = 5 sections, 1 μm each) of the tongues from the same mice identified the expression of IP_3_R3-KO-GFP and PLC**β**3 in the taste receptor cells from the CV papillae (n = 3). Scale bar = 10μm.
(TIFF)
