# Supplementary figures and images for: A subset of broadly responsive Type III taste cells contribute to the detection of bitter, sweet and umami stimuli
Source: PLoS Genet. 2020 Aug 13;16(8):e1008925. doi: 10.1371/journal.pgen.1008925 (PMC7425866; doi:10.1371/journal.pgen.1008925)

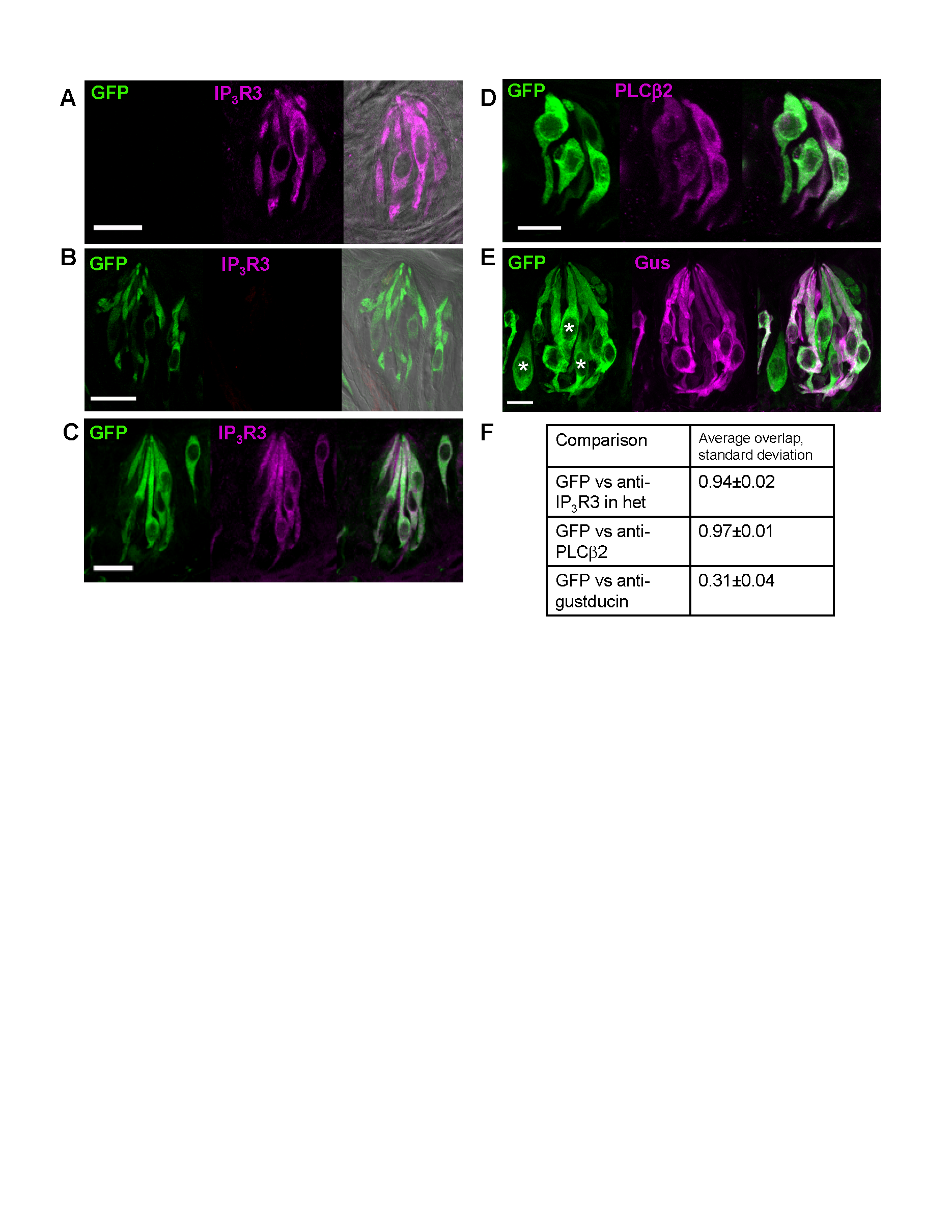

Supplement: S1 Fig — A) Laser scanning confocal micrographs (LSCMs, stack of 5 slices, 1μm each) from WT mice identified anti-IP3R3 labeling in taste receptor cells from the CV (n = 3). B) IP3R3-KO mice express GFP in lieu of IP3R3 and were not labeled by anti-IP3R3 (n = 6). C) LSCMs of the IP3R3-het mouse identified strong co-localization between anti-IP3R3 labeling and GFP expression. D) Anti-PLCβ2 labeling in IP3R3-KO mice found that PLCβ2 co-localizes with GFP, indicating that IP3R3-KO-GFP is specific to Type II cells (LSCMs: stack of 5 slices, 1μm each; n = 4). E) α-gustducin is present in a subset of the IP3R3-KO-GFP taste cells in the CV (LSCMs: stack of 10 slices, 1μm each; n = 3). Asterisks identify some GFP expressing cells that do not express gustducin. Scale bars = 20 μm. F) Co-localization analysis identified the average (± standard deviation) overlapping expression for each target protein with the GFP expression, n = 3 for each. (TIF) [file pgen.1008925.s001.tif]

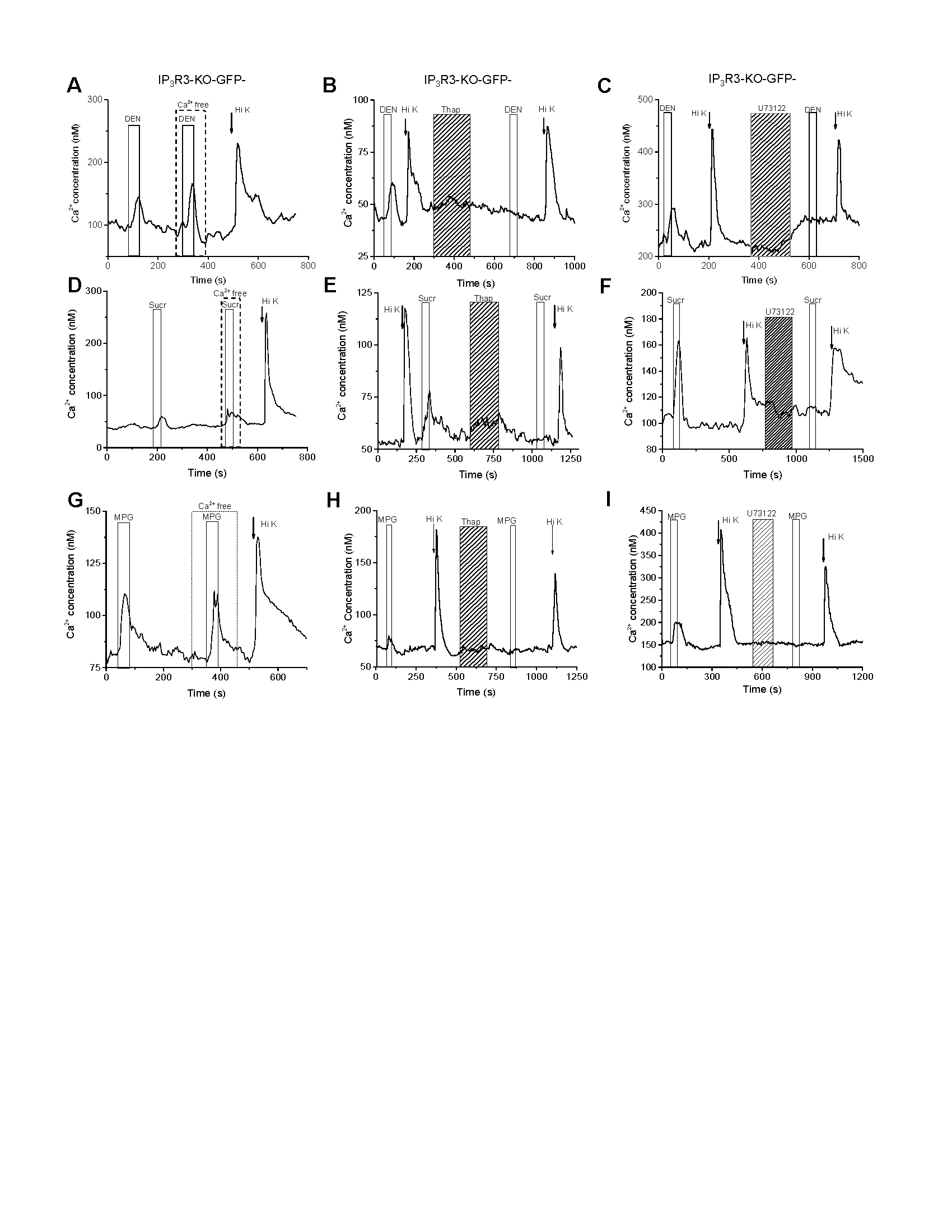

Supplement: S3 Fig — Representative data related to Fig 4. Open columns represent the time that the taste stimulus is presented (40s). The application of Ca2+ free Tyrode’s is indicated by the dashed lines. The stimulus presented during this time is also in Ca2+ free Tyrode’s. The gray hatched columns represent the application of either thapsigargin (Thap) or U73122, both of which are irreversible inhibitors. A) Bitter-evoked taste responses (5mM Den) persist in the absence of extracellular calcium (Ca2+-free) and are abolished by the SERCA pump inhibitor thapsigargin (B) as well as the PLC blocker U73122 (C). D) Responses to sweet stimuli (20mM sucralose, Sucr) persist in Ca2+-free and are abolished by thapsigargin (E) and U73122 (F). G) Umami stimuli (10mM MPG) persist in Ca2+-free and were abolished by thapsigargin (H) and U73122 (I). (TIFF) [file pgen.1008925.s003.tiff]

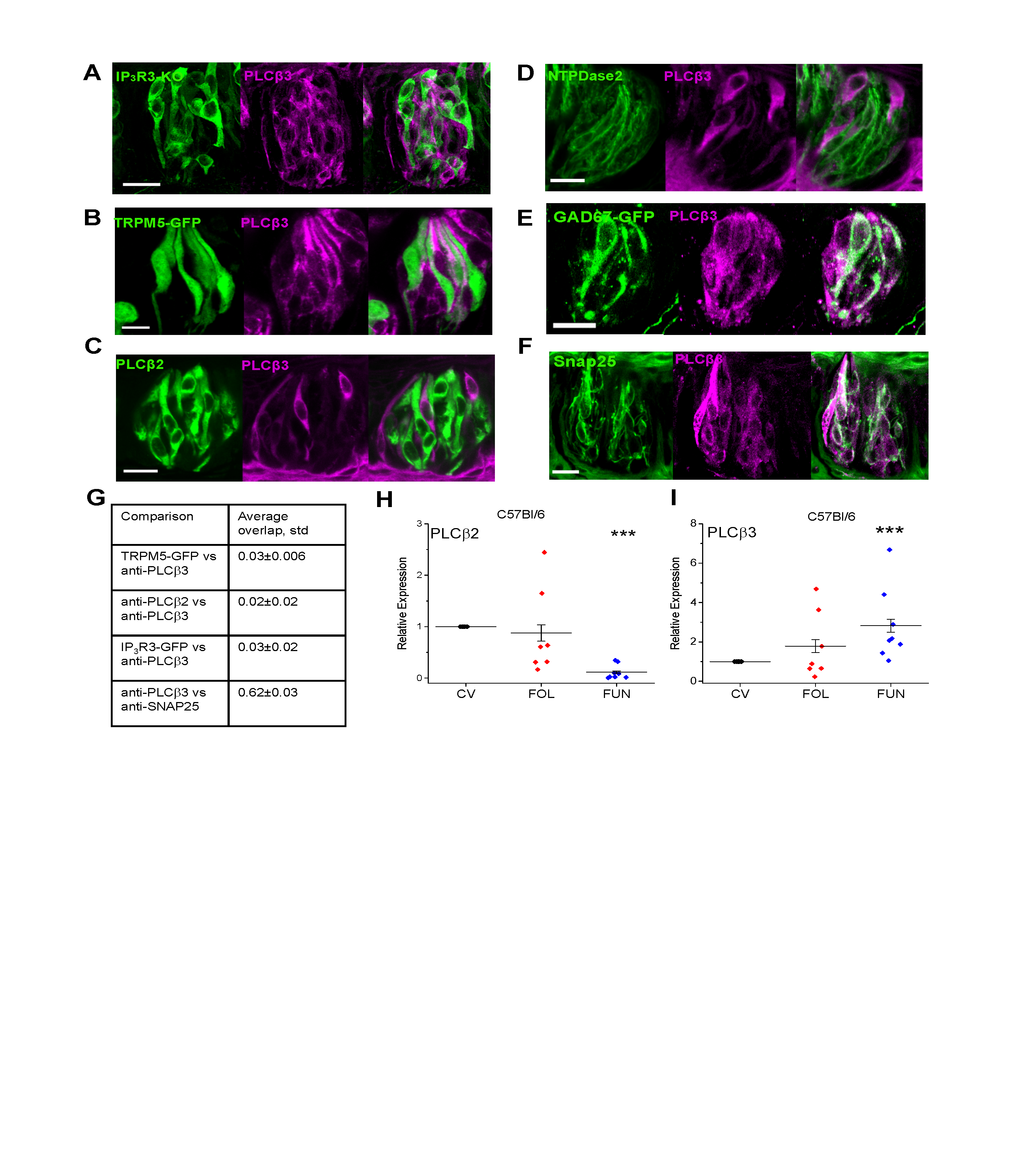

Supplement: S4 Fig — A) Laser scanning confocal micrographs (LSCMs, stack of 5 slices, 1μm each) of PLCβ3 immunostaining in the IP3R3-KO-GFP mice reveal that PLCβ3 is expressed in a separate population from the GFP positive taste cells in the CV. B) Anti-PLCβ3 labeling in the CV of TRPM5-GFP mice determined that PLCβ3 is expressed in taste cells lacking GFP expression (LSCMs: stack of 5 slices, 1μm each; n = 4). C) Co-labeling with anti-PLCβ2 and anti-PLCβ3 in the CV of C57BL/6 mice revealed that these PLCβs are expressed in separate taste cell populations (LSCMs: stack of 5 slices, 1μm each; n = 3). D) Co-labeling with anti-NTDPase2 and anti-PLCβ3 in the CV of C57BL/6 mice determined that these markers are expressed in separate taste cell populations (LSCMs: stack of 5 slices, 1βm each; n = 3). Scale bar = 20μm. E) Anti-PLCβ3 labeling in the GAD67-GFP mice determined that PLCβ3 is partially expressed in taste cells with GFP expression (LSCMs: stack of 5 slices, 1μm each; n = 4). F) Immunohistochemical analyses (LSCMs: stack of 5 slices, 1μm each) using anti-PLCβ3 and anti-SNAP25 revealed some co-localization between PLCβ3 and SNAP25 in CV papillae. Scale bars = 10μm. G) Co-localization analysis identified the average (± standard deviation) overlapping expression for PLCβ3 with TRPM5-GFP, anti-PLCβ2, IP3R3-GFP, or anti-SNAP25 expression, n = 3 for each. mRNA was isolated from taste cells originating in the different papillae types from C57BL/6 mice. Taste cells were analyzed from at least five different mice for each. Values were normalized to GAPDH expression and are presented as a ratio to values from the CV papillae for (H) PLCβ2 and (I) PLCβ3. (***, p<0.001). (TIFF) [file pgen.1008925.s004.tiff]

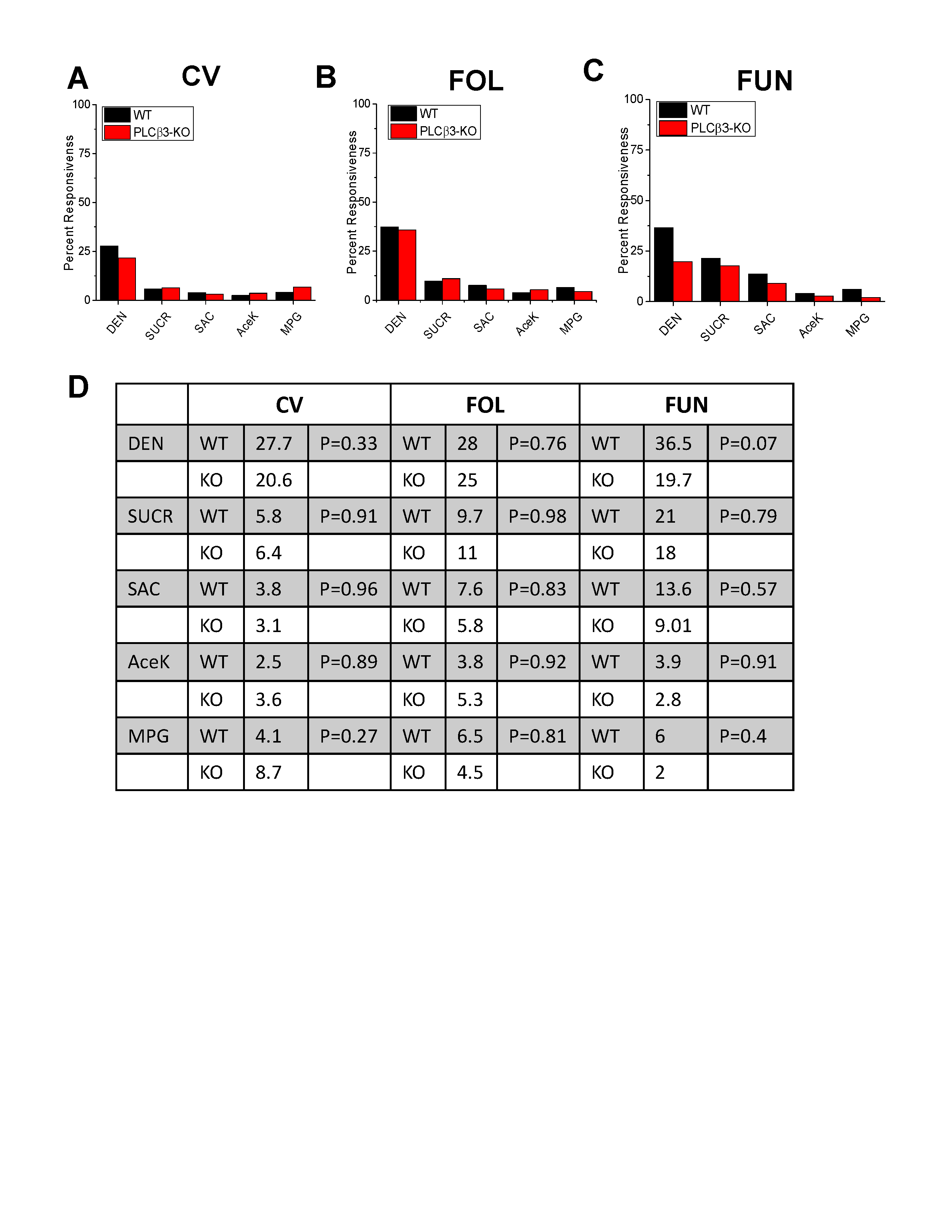

Supplement: S5 Fig — Chi square analysis with Yate’s correction for continuity was used to compare the response rate or frequency of evoked Ca2+ responses to different taste stimuli between wild type (black bars) and PLCβ3-KO (red bars) mice for taste cells from CV (A), Fol (B), and Fun (C) papillae. D). Table of the stimulus response rate for each papillae type in WT and KO mice. P values for each comparison are also shown. No significant differences were found for any of the comparisons. (TIFF) [file pgen.1008925.s005.tiff]

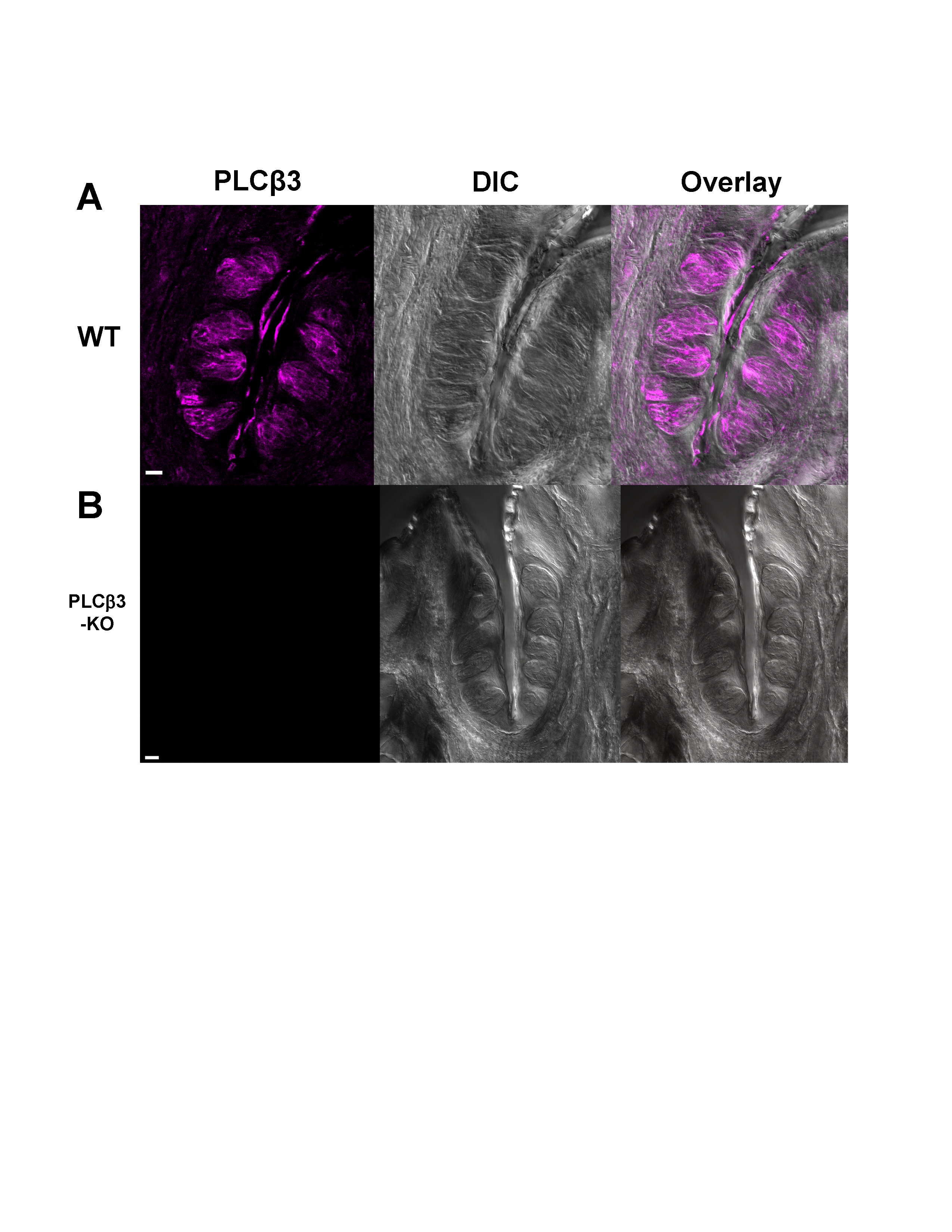

Supplement: S6 Fig — A) LSCMs (stack of 5 slices, 1μm each) from WT mice identified anti-PLCβ3 labeling in taste receptor cells from the CV (n = 3). B) CV taste cells from the PLCβ3-KO mice were not labeled by anti-PLCβ3 (n = 3). Scale bar = 10μm. (TIF) [file pgen.1008925.s006.tif]

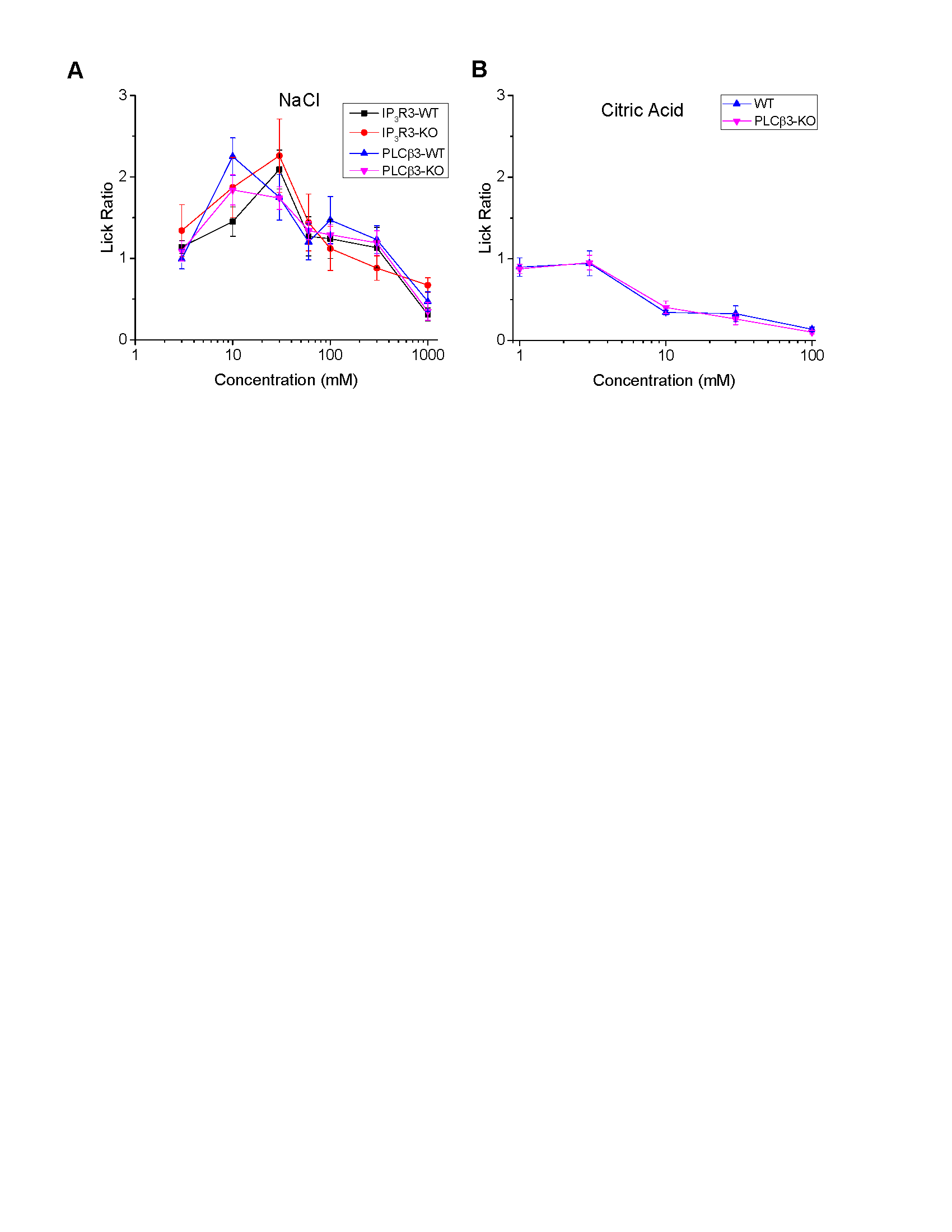

Supplement: S7 Fig — Average lick data (±standard deviation) from brief-access behavioral tests compare the responses of IP3R3-KO (red line) and PLCβ3-KO (pink line) to WT (IP3R3-WT, black line; PLCβ3-WT, blue line). A) No significant differences were detected between the WT and KO mice for any concentration of NaCl (0, 3, 10, 30, 60, 100, 300, 1000mM) tested. B) No significant differences were detected between the PLCβ3 WT (blue line) and KO (pink line) mice for any concentration of citric acid (0, 1, 3, 10, 30, 100mM) tested. For all experiments, 5 mice of each genotype were used. Data were compared by repeated measures ANOVA. Significant interaction terms were followed by Tukey’s Honestly Significant Difference tests. (TIF) [file pgen.1008925.s007.tif]

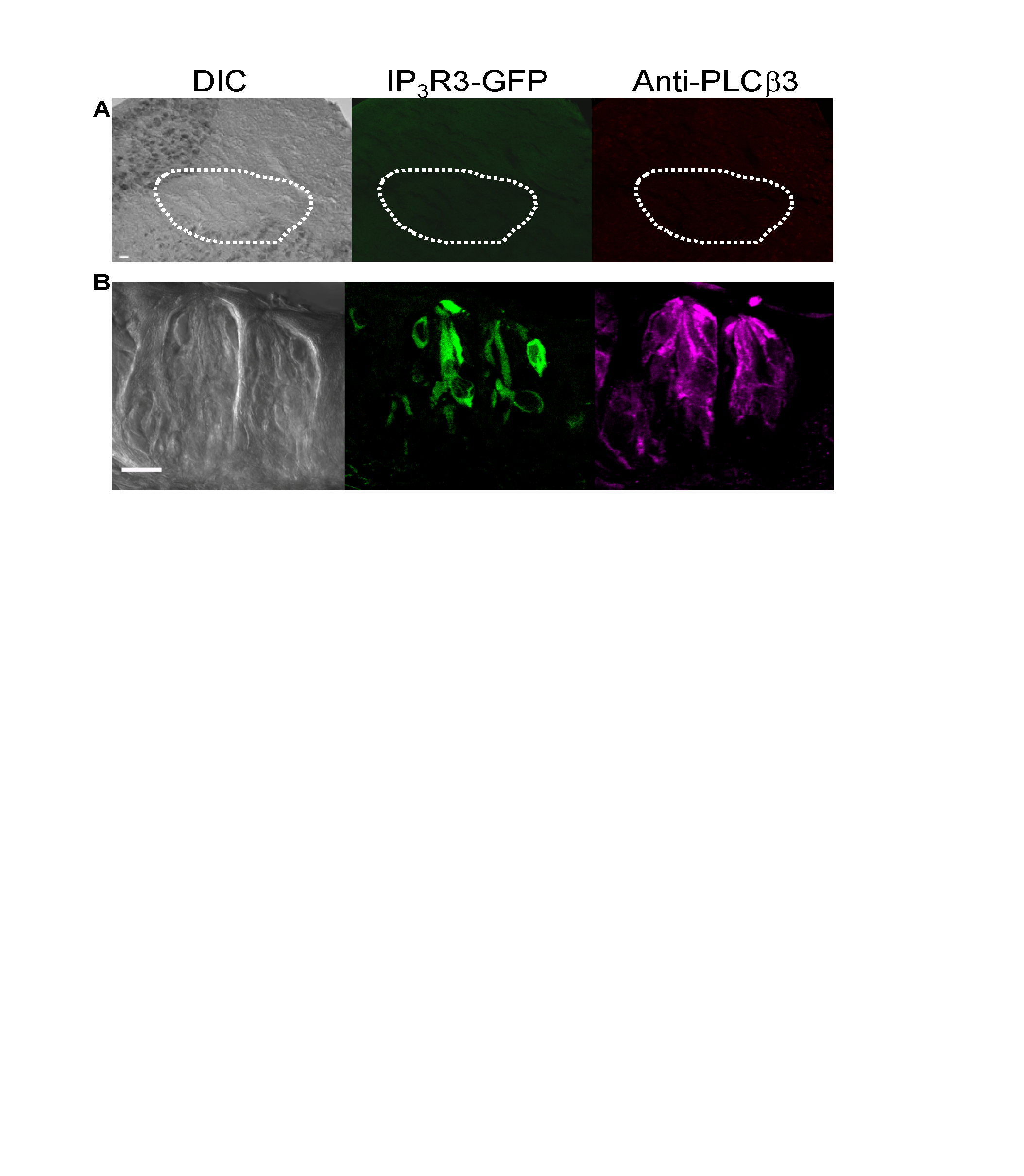

Supplement: S8 Fig — CV papillae and IRNTS sections were evaluated in parallel. A) Analyses of the brain sections from the IP3R3-KO mice revealed no GFP labelling in the IRNTS. Immunohistochemical analysis using anti-PLCβ3 also did not detect any PLCβ3 expression. Scale bar = 50μm. B) LSCM analyses (n = 5 sections, 1 μm each) of the tongues from the same mice identified the expression of IP3R3-KO-GFP and PLCβ3 in the taste receptor cells from the CV papillae (n = 3). Scale bar = 10μm. (TIFF) [file pgen.1008925.s008.tiff]
